# Experimental investigation of the spatial movement law of straw–root–soil complexes during slope contour rotational tillage operations based on the tracer method

**DOI:** 10.3389/fpls.2026.1862550

**Published:** 2026-07-08

**Authors:** Mingzhe Lv, Jingbin Sun, Yuda Lu, Gang He, Lijing Liu, Jing Ying, Yuying Zhang, Yingkai Chen

**Affiliations:** 1 College of Mechanical and Automotive Engineering, Liaocheng University, Liaocheng, China; 2 State Key Laboratory of Agricultural Equipment Technology, Beijing, China; 3 Chinese Academy of Agricultural Mechanization Sciences Group Co., Ltd., Beijing, China; 4 Key Laboratory of Agricultural Equipment Technology for Hilly and Mountainious Areas, Ministry of Agriculture and Rural Affairs, Chengdu, China; 5 Sichuan Academy of Agricultural Machinery Sciences, Chengdu, China; 6 Shandong Academy of Agricultural Sciences, Jinan, China

**Keywords:** hilly and mountainous areas, migration characteristics, rotary tillage on slope land, straw-root-soil complexes, tracer method

## Abstract

During contour rotary tillage on sloping terrain, the migration characteristics of the straw–root–soil complex directly affects the effectiveness of straw return and soil sustainability, but the underlying mechanisms remain unclear, hindering the development of specialized rotary tillage equipment. This study uses a self-developed bench test platform for rotary tillage on sloped terrain in hilly and mountainous regions and tracer method to systematically investigate the effects of slope gradient (5°, 10°, 15°), blade shaft rotational speed (200–300 r/min), forward farming speed (0.2–1.0 km/h), and straw content (0.4–1.2 kg/m²) on the migration characteristics of soil aggregates. The test results demonstrate that the forward farming speed is the most effective controllable parameter for suppressing the horizontal and lateral displacement of soil complexes. Increasing the speed from 0.2 km/h to 1.0 km/h can reduce the lateral displacement of straw by 65.3%. An increase in the rotational speed of the blade shaft intensifies the migration of the complex mass, with a notably increased displacement toward the downhill side under steep slope conditions (15°). The slope gradient is the dominant natural factor driving the asymmetric movement of the complex along the lower side of the slope. When the slope increases from 5° to 15°, the lateral displacement of the straw increases by 166%. Straw mulching effectively mitigates tillage-induced soil erosion. When the straw content increases from 0.4 kg/m² to 1.2 kg/m², the lateral soil displacement is reduced by 37.7% to 51.9%. Through orthogonal experiments and response surface analysis, the hierarchical order of the factors influencing soil complex transport was determined. In terms of soil lateral displacement, the primary influencing factor is the slope gradient. With respect to soil horizontal displacement, the dominant factor is the forward farming speed. Corresponding regression prediction models were developed. This study provides the first systematic evaluation of the transport mechanisms governing the straw–root–soil complex on sloped terrain under contour rotary tillage conditions, thereby establishing a theoretical foundation for designing specialized tillage equipment for hillside agriculture and performing quality control in the incorporation of crop residue.

## 1 Introduction

Slope agriculture plays a pivotal role in global food security and ecological equilibrium, particularly in countries such as China, where hilly and mountainous terrain constitutes approximately 70% of the national land area (Sun et al., 2023). The production efficiency directly influences regional economic development and rural revitalization. However, owing to natural constraints such as rugged terrain and significant slope variations, the mechanization level of slope land agriculture has substantially lagged, with the operational efficiency being less than 60% of that in plain areas (Zeng et al., 2026). This is often accompanied by exacerbated soil erosion and degradation of topsoil quality. The operational quality and sustainability of rotary tillage equipment, a primary tillage method employed on sloping land, directly affects soil conservation. Straw incorporation is a critical measure for increasing soil organic matter content and improving soil structure. However, under sloping field conditions, the movement behavior of the straw–soil complex during rotary tillage significantly differs from that observed on level ground. The complexity of tillage operations on slopes is markedly increased because of the gravitational component acting along the inclined plane, variations in soil stability, and the dynamic interactions among the machinery, soil, and straw residues. Under such conditions, the kinematic behavior of the straw-soil complex during rotary tillage differs considerably from that observed on level terrain. Academic research has provided preliminary findings regarding straw incorporation and rotary tillage operations, including the development of straw–root–soil complex structures, discrete element modeling, and hillslope cultivation experiments.

In straw–root–soil complex modeling, the structural characteristics of the stalk–root–soil complex, which serves as the direct target in slope land rotary tillage operations, constitute the basis for investigating the mechanisms of soil–tool interactions. In terms of physical characterization, scholars have conducted multidimensional investigations. Zhou et al. (2020) employed three-dimensional coordinate measurement technology to analyze the distribution patterns of straw in soil following the operation of different rotary tillage and subsoiling straw-return machines, thereby establishing a methodological framework for quantifying the spatial distribution of straw residues. Cherubin et al. (2021) proposed a remolding method for soil with high straw content by placing sugarcane straw into compacted soil samples, and verified the “damping effect” of straw coverage on soil. De Lima et al. (2018) investigated the effects of initial bulk density and matric suction on the compression characteristics of no-till soils, establishing a predictive model for compaction risk assessment. Wang et al. (2025) conducted direct shear tests on straw-soil complexes to evaluate the influence of straw content on soil shear strength, and explored the soil–tool–straw interaction mechanisms using the discrete element method, thereby offering valuable insights for the simulation modeling of complex materials. Faloye et al. (2021) elucidated that effective stress serves as a critical parameter for predicting the mechanical behavior of soil, and investigated the influence of soil texture, bulk density, and other relevant factors on its magnitude. By utilizing field-measured data and employing a discrete element–multibody dynamics coupling methodology, Lin et al. (2024) developed a simulation model for characterizing the interaction between straw residues and soil. In the realm of root-soil synergy, scholars (Zhang et al., 2023b) have advanced various theoretical models—including those based on shear displacement, root bundle reinforcement, and energy methods—offering diverse frameworks for the mechanical characterization of root-soil complexes. Furthermore, Sun et al. (2022a) investigated the transmission mechanism of soil compaction stress under contour tillage on sloping land by employing the discrete element method in con-junction with field experiments. An et al. (2022) demonstrated through consolidation tests that the incorporation of straw significantly enhances the rebound and compressive characteristics of soil. The aforementioned research has laid a critical foundation for the structural characterization and mechanical modeling of the straw-root-soil complex. However, under slope-specific conditions, the dynamic reconstruction of the complex and its coupled response mechanism with slope gradient remain to be further elucidated.

Regarding the interaction mechanism between soil-engaging components, soil, and crops, Cheng et al. (2021) utilized the Discrete Element Method (DEM) to simulate the adhesion behavior of wet clay to tillage tools during paddy field rotary tillage, thereby providing guidance for the calibration of soil parameters. Ucgul and Saunders (2020) validated the reliability of the Discrete Element Method (DEM) in simulating soil-tool interaction through a comparison between simulation and field experiments; Zeng et al. (2020) established a discrete element model to simulate the interaction between soil, agricultural implements, and straw residues, and validated the model through simulations and soil-bin tests using four distinct types of shovel tools. Torotwa et al. (2021), grounded in the principles of bionics, investigated the interaction mechanisms be-tween a biomimetic disc and straw–soil complexes, thereby offering novel insights for the structural optimization of cutting tools. Xie et al. (2026) conducted a comprehensive investigation on cinnamon soil, employing an integrated simulation-experimental methodology to calibrate the contact parameters between rotary tillage blades and soil. Chen et al. (2024a) developed a complex soil-straw model based on the distribution characteristics of straw residues, which effectively predicts the mechanical interactions between the model and soil-engaging components. Sun et al. (2022b) calibrated and validated the discrete element parameters for the interaction between slope soil and rotary tillage components, providing reliable parameters for the simulation of machine-soil interactions. Zhang et al. (2023a) established a rotary tiller-soil-straw interaction model to analyze key factors influencing power consumption and tillage quality. Investigators (Xu et al., 2022a, b, 2021, 2022c) systematically examined the influence of operational parameters on straw displacement and burial efficacy through a specialized soil-bin test platform, thereby proposing quantitative methodologies for straw distribution analysis. This research furnishes a theoretical foundation for optimizing the quality of straw incorporation.

In the realm of experimental research on tillage erosion on slopes, relevant studies have primarily focused on the impact of tillage practices on sustainable soil utilization. Sun et al. (2023) elucidated research advancements concerning agricultural machinery-induced soil compaction and tillage erosion on sloping terrain, while also summarizing computer-aided methodologies for the design of mountain agricultural machinery and the study of interaction mechanisms between such machinery and soil. Madarász et al. (2021) experimentally demonstrated that protective tillage significantly enhances soil erosion resistance. Wang et al. (2022) investigated the inhibitory effect of root–soil complexes on hoeing-induced erosion on sloped land using magnetic tracer technology. Yu et al. (2024) built an experimental platform to reveal the advantages of contour farming in improving soil texture and reducing nutrient loss. Gristina et al. (2022) employed the colored sand tracer technique to establish a regression model relating slope gradient to soil migration, thereby providing a methodological reference for predicting hillslope erosion. Sun et al. (2022c) systematically elucidated the soil erosion patterns in contour tillage operations on sloping terrain through integrated discrete element simulations, soil bin experiments, and field trials on hillslopes. Their research provides methodological support for investigating the interactions within the agricultural machinery-soil-crop system under slope conditions. Xia et al. (2026) investigated the effects of rotary tillage combined with straw return on soil erosion using X-ray CT scanning technology. The study revealed that straw return significantly enhances soil macroporosity, hydraulic radius, and pore connectivity under different tillage practices. Zhao et al. (2016) employed a physical tracer method to quantitatively analyze soil displacement induced by moldboard plowing on typical slopes in the black soil region of Northeast China. The results revealed a significant positive correlation between slope gradient and the magnitude of tillage-induced displacement, with severe erosion observed at convex backslopes, shoulder slopes, and steeper areas. This study demonstrates that even in black soil regions with mild slopes, intensive tillage operations characterized by deep penetration and high speed lead to substantial erosion. The findings offer valuable insights for understanding tillage erosion patterns across diverse topographic conditions.

Although progress has been made in characterizing soil complexes and understanding the mechanism of rotary tillage on sloping land, current research remains limited in terms of the following aspects. First, existing studies have focused predominantly on individual soil components, and systematic observations of the motion trajectories and mechanical responses of straw–root–soil complex during rototilling on sloping terrain are lacking. Additionally, the dynamic mechanisms governing the multifactor coupling effects of the implementation parameters, complex system variables, and terrain slopes remain inadequately characterized, thereby impeding the precise design and optimization of specialized rotary tillage operations for sloped terrains. To address these research gaps, this study focuses on rotary tillage operations following corn harvesting on sloping terrain. Leveraging field-measured slope data, a slope–characteristic-integrated straw–root–soil complex model is constructed. Through bench testing of sloping-land rotary tillage operations, combined with high-speed imaging and tracer techniques, the spatial translocation behavior of the soil complex under slope conditions is precisely characterized from both dynamic and static perspectives. Additionally, the influence of the slope gradient, blade shaft rotational speed, the forward farming speed, and straw incorporation rate on the migration trajectory and distribution characteristics of the complex bodies are systematically analyzed. Through orthogonal experiments and regression modeling, the primary and secondary influences of various implementation factors and slope conditions on the displacement of soil complex components are delineated, and an optimal parameter configuration for rotary tillage operations on sloping terrain is proposed. The integration of complex soil remodeling on sloped land with bench test validation experiments will provide robust support for advancing conservation tillage technologies and equipment development in hilly and mountainous regions.

## 2 Materials and methods

### 2.1 Working principle of the test platform

#### 2.1.1 Overall structure

**Figure 1:**
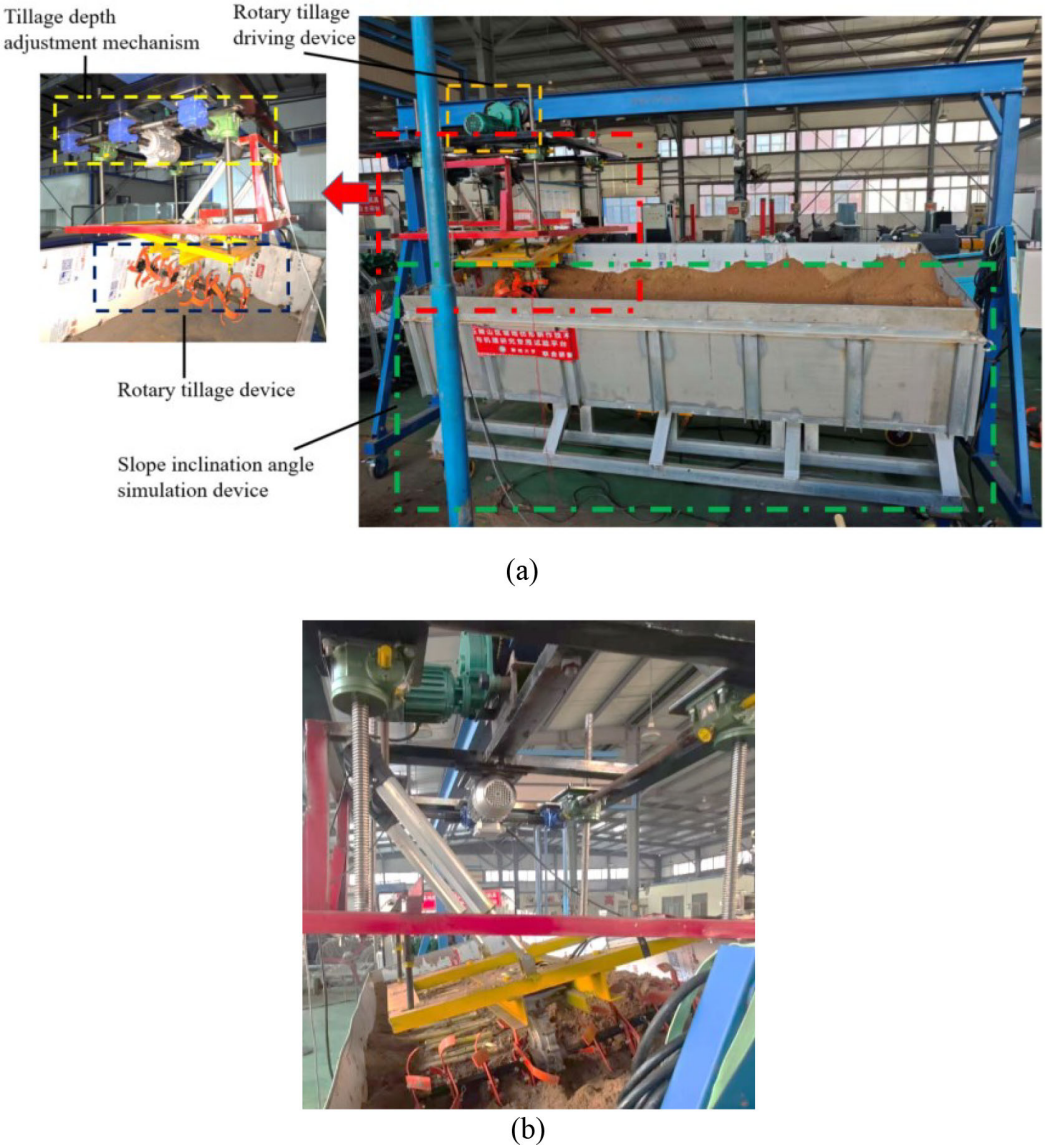
Working principle of the specialized rotary tillage test platform for sloping land in hilly and mountainous areas. **(a)** The overall structure diagram of the machine; **(b)** The Side view of the rotary tillage test bench simulating a slope of 10°.

To elucidate the spatial transport dynamics of straw–root–soil complexes under sloped land conditions, in this study, systematic bench tests were conducted with a self-developed rotary tillage test platform specifically designed for hilly and mountainous slope terrain (as shown in **Figure 1**) (Sun et al., 2025). The core advantages of this platform are as follows: it can randomly simulate the slope angle, automatically shape the rotary tiller shaft, precisely control the tillage depth and infinitely adjust the forward farming speed. This provides controllable experimental conditions for the comprehensive study of the interaction mechanism of the machine–soil complex under rotary tillage conditions on slopes.

The structural parameters of the machine are presented in **Table 1**. The apparatus is capable of simulating a maximum slope gradient of 20°. It allows for a continuously adjustable forward farming speed ranging from 0 to 3 km/h, and it’s the blade shaft rotational speed can be continuously adjusted from 0 to 300 r/min. Furthermore, the adjustable tillage depth ranges from 0 to 30 cm. These functionalities collectively fulfill the experimental requirements for multifactorial and multilevel investigations.

**Table 1:** Structural parameters of the whole machine.

Parameters	Numerical value
Overall machine quality/kg	800
Number of electric actuators/pieces	2
Number of hydraulic cylinders/pieces	2
Length × Width × Height/(mm × mm × mm)	4000×1200×2500
Number of universal wheels	4
Simulated slope inclination range/(°)	0~20 (Continuous adjustment)
Rotary tiller forward farming speed/(km·h^-1^)	0~3 (Continuous adjustment)

#### 2.1.2 Working principle

Traditional rotary tillage implements are suitable for relatively flat plains; however, when these implements operate on steeper slopes and undulating terrains typical of hilly and mountainous regions, they exhibit poor adaptability to sloped terrains and have difficultly maneuvering. This study aims to investigate the transportation patterns of straw–root –soil complexes in sloping fields using a self-developed specialized test platform for hillslope rotary tillage operations. The specialized testing platform is primarily composed of a rotary tillage travel guidance device, rotary tillage propulsion unit, rotary tillage depth adjustment mechanism, profiling device for tillage operations, rotary tillage operational assembly, and slope inclination simulation module. The rotary tillage traveling device is powered by a 380 V three-phase asynchronous motor, which propels the tillage operating mechanism along the beam of the guidance system. The tillage depth is regulated by the lifting adjustment device, while the rotary tillage motor drives the rotation of the tillage blade shaft to perform the tillage operation. The slope inclination simulation device is capable of replicating various gradient conditions, while the profiling mechanism for rotary tillage operations automatically adjusts the tilt angle of the blade shaft to align with the slope surface, thereby enabling terrain-adaptive operation on sloping fields.

Operating principle of the experimental platform: First, a manual hydraulic pump is employed to extend the hydraulic cylinder, and the soil trough platform is tilted to the target slope. The profiling device for rotary tillage operations utilizes an electric actuator to control the rotation of the tillage inclination plate around the axis of the lifting plate, enabling dynamic alignment of the blade shaft inclination with the soil trough slope. The screw lifting mechanism is subsequently engaged to vertically adjust the tillage lifting plate to the predetermined working depth. The rotary tillage motor transmits power to the rotary blade shaft via a speed reducer, resulting in the operation of the rotary tillage blade set. Simultaneously, the propulsion drive motor of the tillage system is engaged, and the tillage platform moves forward along the contour of the sloped terrain. This process faithfully replicates dynamic rotary tillage operations under practical sloped land conditions.

**Figure 2:**
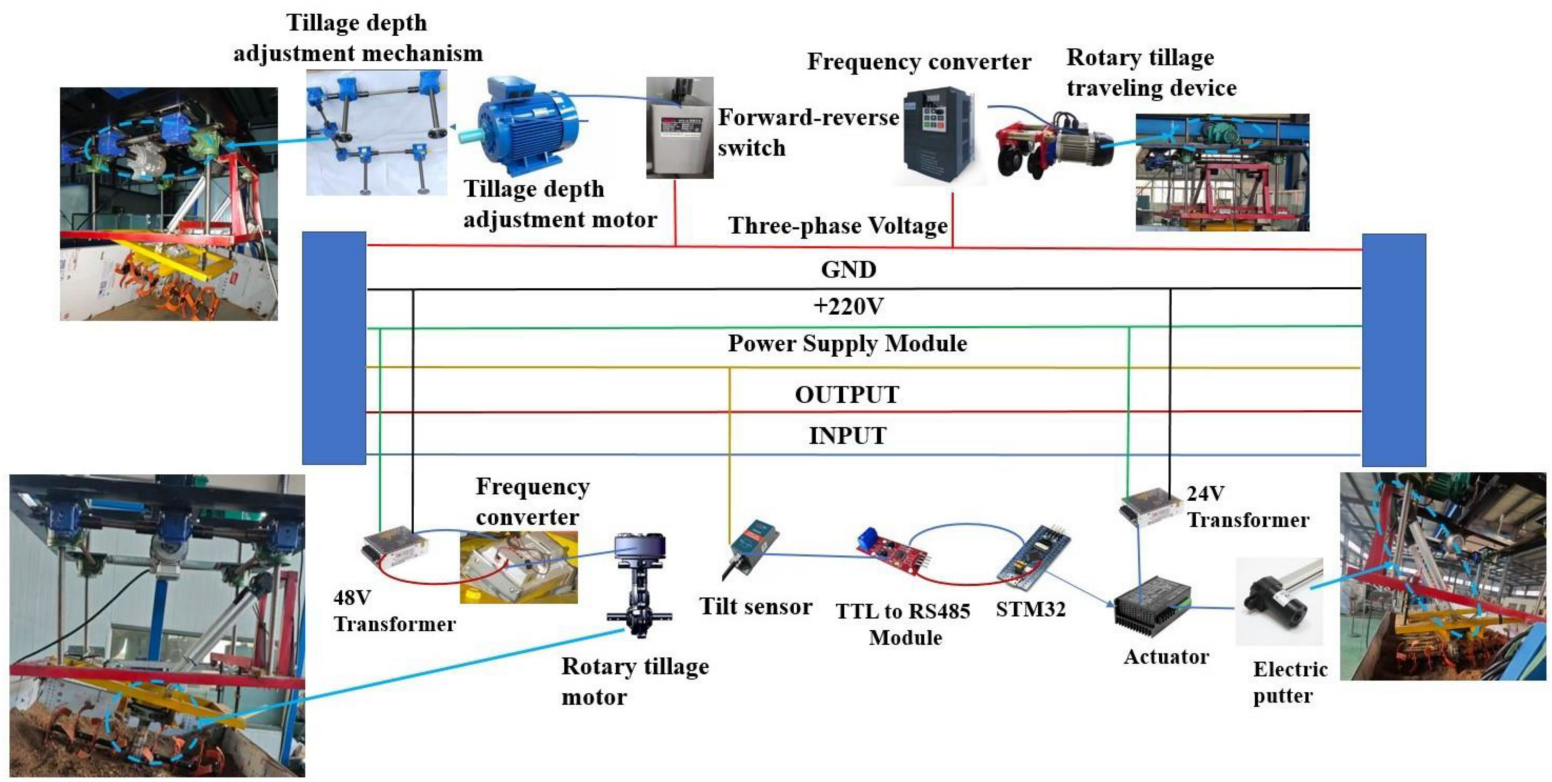
Control system for the rotary tillage experimental platform on sloping fields.

The main objective of the control system module is to synchronize the soil bin inclination mechanism and the rotary tillage tilt adjustment device. This integration aims to enable the contour-following operation of the rotary tillage components when the inclination simulation apparatus emulates any slope condition within the range of 0° to 20°. The hardware configuration and signal flow of the control system implemented via the experimental platform are shown in **Figure 2**. The driving motor for the rotary tillage system is a three-phase asynchronous motor with a rated power of 1.5 kW. Through inverter control, the speed can be regulated within the range of 0 to 3 km/h, thereby complying with the requirements of the Chinese national standards for rotary tillers. The rotary tillage motor of the test platform is driven by a frequency converter. The target blade shaft rotational speed is preset according to the test scheme, and the output frequency of the frequency converter is adjusted to make the blade shaft rotational speed of the rotary tillage motor reach the set value. Before the formal test, a contact−type tachometer is used to measure the actual blade shaft rotational speed at multiple points under both no−load and load conditions, thereby ensuring that the actual blade shaft rotational speed during operation complies with the preset value. The AE200 inverter model (three-phase 380 V, 0.5 kW power rating) provides overvoltage, under voltage, and overcurrent protection. Additionally, this inverter can control the motor of the rotary tillage driving device to enable both forward and reverse rotations, thereby facilitating forward and reverse operations of the rotary tillage driving device. A three-phase asynchronous motor is selected for the tillage depth adjustment mechanism of the rotary tillage unit. The motor, which is designed to bear loads of up to 1 ton, is regulated via a forward–reverse switch to achieve bidirectional rotation. As a result, the screw lifting rod can be moved in either direction, thereby enabling vertical adjustment of the tillage depth.

The inclination of the soil trench is simulated by extending the hydraulic cylinder through a specified stroke, and an inclination sensor detects the tilt signal. The system, which is developed based on an STM32 microcontroller, is powered by a power supply module (converting 220 V to 24 V/48 V) to power all the actuating mechanisms. The controller receives slope angle signals from an inclinometer (via an RS485 module) and velocity feedback signals from an encoder. After these inputs are processed through the internal decision-making algorithm, the controller actuates the electric linear actuator via a relay module to ensure that the tillage angle is consistently aligned with the slope inclination. The entire control process involves closed-loop regulation of the slope gradient, blade shaft rotational speed, and tillage depth, thereby ensuring the operational capability of the profiling platform under various slope conditions.

### 2.2 Analysis of the forces exerted on straw under sloping land rotary tillage conditions

**Figure 3:**
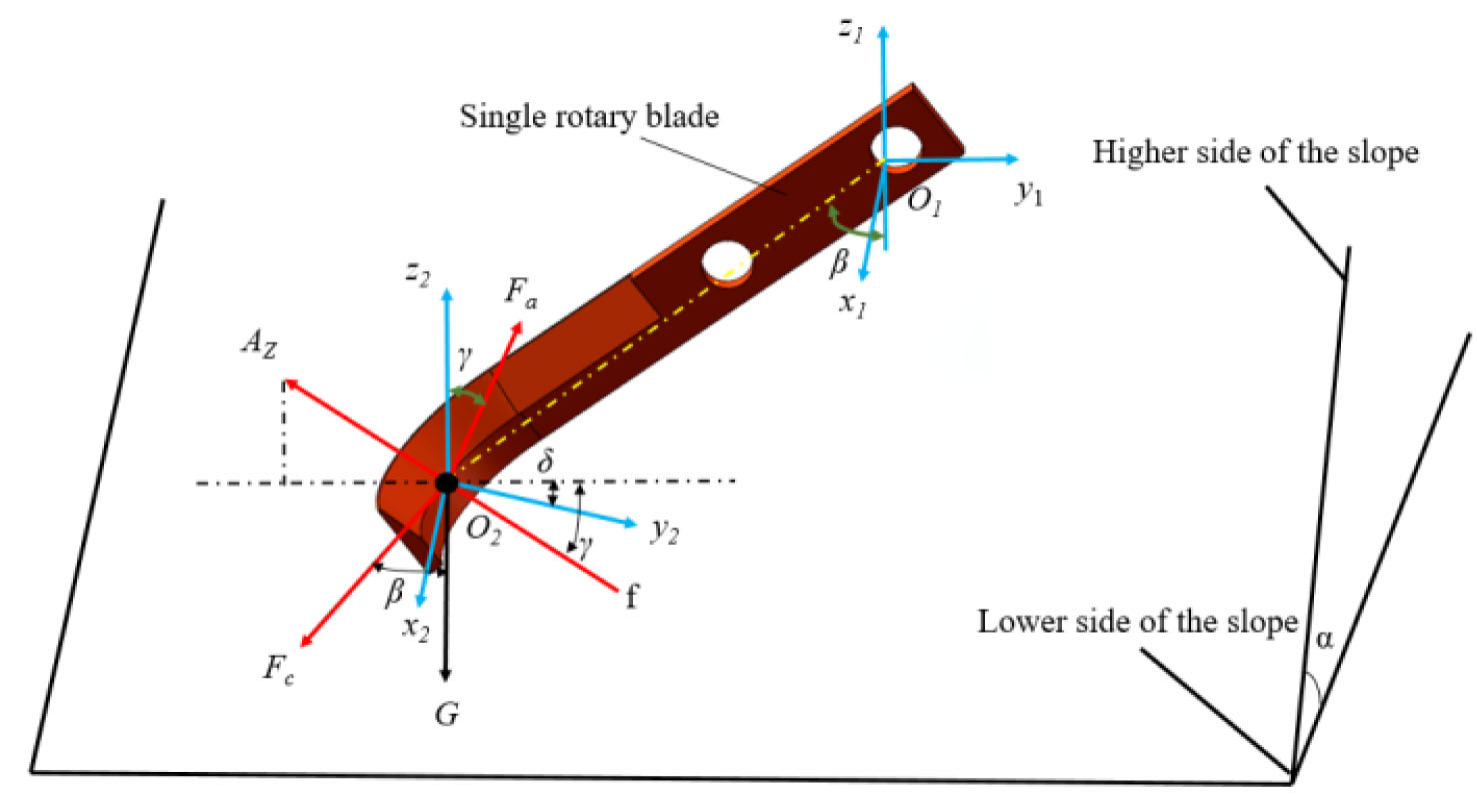
Spatial force analysis of straw.

**Figure 4:**
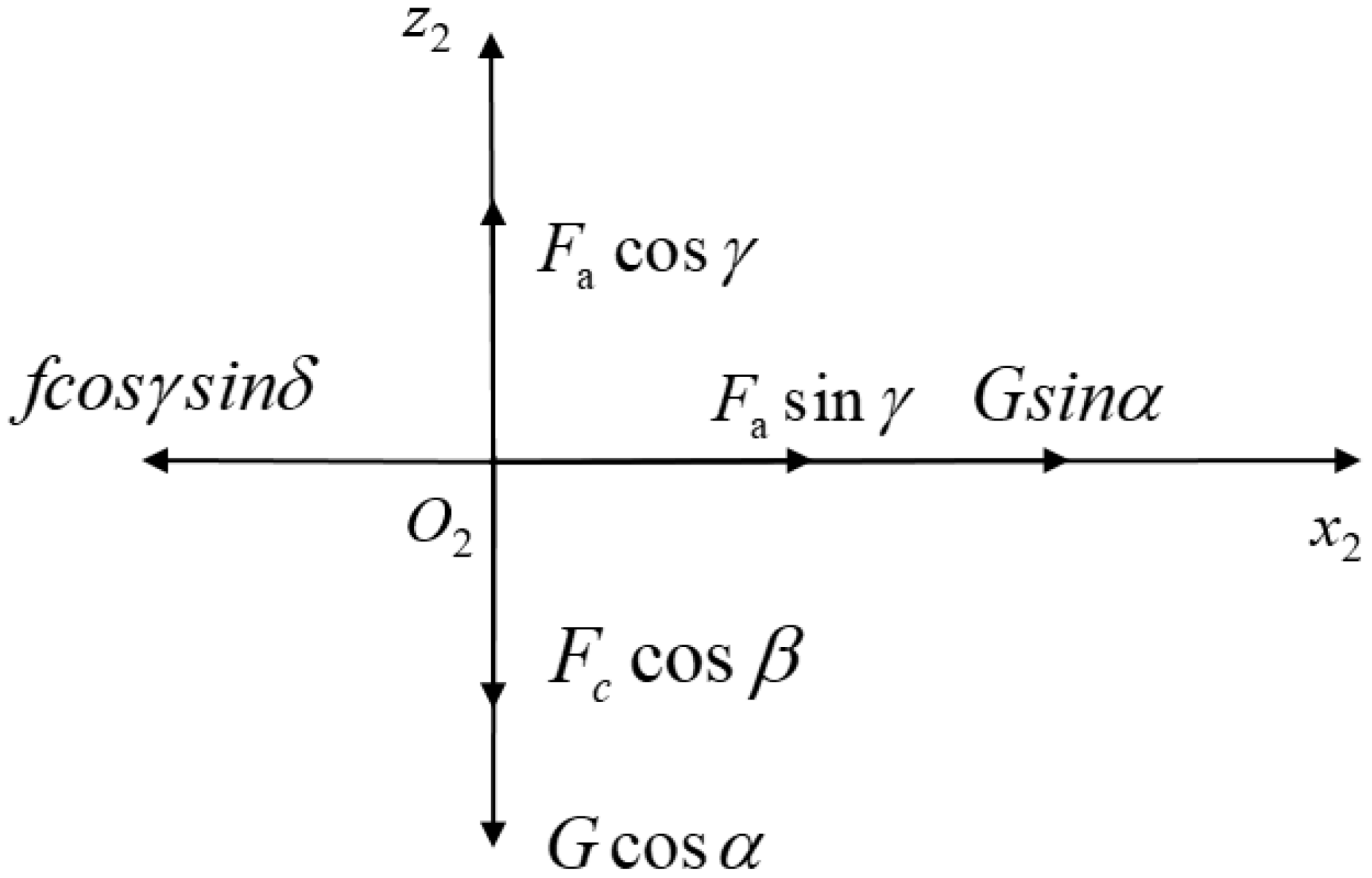
Spatial force analysis of straw under the action of rotary tillage blades.

To elucidate the mechanisms governing straw motion during rotary tillage operations on slopes, a theoretical analysis of the forces acting on the straw units under the influence of the tillage blades is presented. Assuming that the straw is a rigid body at the moment it leaves the tangential plane of the rotary tiller blade and neglecting its bending deformation and interaction with the surrounding straw, a force model is established, as shown in **Figures 3**, **4**.

During contour rotary tillage operations on sloped terrain, the central axis of the rotary tiller blade consistently remains parallel to the slope surface. The hillslope angle is set as *α*, and a spatial Cartesian coordinate system is established, with the intersection point of the central plane of the tool holder and the central axis of the cutter shaft as the origin. The direction of the cutter shaft central axis (parallel to the slope surface) is defined as the *x*_1_-axis, the forward direction of the working implement is defined as the *y*_1_-axis, and the direction perpendicular to the slope surface is defined as the *z*_1_-axis.

The coordinate system was translated to the centroid *O_2_* of the straw unit, resulting in the new coordinate system *x_2_O_2_y_2_*. At the moment when the straw detaches from the orthogonal plane of the rotary blade, it is subjected to the following forces: the normal force *F_n_*, the frictional force *f*, the gravitational force *G*, and the centrifugal force *F_c_*. By resolving these forces into the *x_2_O_2_y_2_* plane (as illustrated in **Figures 3**, **4**), the resultant force acting on the straw along the *x_2_* direction (i.e., the lateral direction of the slope) can be expressed, as shown in Equation 1.

(1) $$F_{x}=\frac{F_{c}\cos\beta +Gcos\alpha }{cos\gamma }sin\gamma +Gsin\alpha -fcos\gamma sin\delta$$

In the formula, *β* represents the rotation angle of the blade shaft when the straw is detached (°);
*γ* represents the angle between the *x_2_O_2_y_2_* plane and the normal cutting edge of the rotary tiller (°);
*δ* represents the angle between the *A_z_* direction of the straw movement projected onto the *x_2_O_2_y_2_* plane and the *y_2_* axis (°).

The rotational speed of the rotary blade shaft is denoted as *n* (r/min). The corresponding angular velocity *ω* (rad/s) is calculated as *ω = 2πn/60*.

Substituting the centrifugal force *F_c_ = mω²r*, the gravitational force *G = mg*, and the frictional force *f = μ (F_c_ cos β + G cos α)* into the above equation, and by using the geometric relationship *γ = θ - π/2*, where *θ* is the bending angle of the cutting edge, the expression for the resultant force of the straw’s lateral movement is obtained, as shown in Equation 2.

(2) $$F_{x}=-(m\omega^{2}rcos\beta +mgcos\alpha )cot\theta +mgsin\alpha -\mu (m\omega^{2}rcos\beta +mgcos\alpha )cos\gamma sin\delta$$

In the equation, *m* denotes the unit mass of the straw (kg);
*r* represents the radius of gyration of the straw’s centroid (mm);
*ω* indicates the angular velocity of the blade shaft (rad/s);
*μ* is the coefficient of friction between the straw and the tangential plane of the rotary blade;
*g* stands for the gravitational acceleration (m/s²).

The mechanical condition for the lateral migration of straw (i.e., along the slope direction) is *F_x_* > 0. As indicated by the preceding equation, the driving force for the lateral movement of straw is jointly influenced by the rotational speed of the blade shaft, the bending angle of the tangential blade, the slope gradient, and the frictional characteristics between the straw and the blade surface. Specifically, as the slope angle *α* increases, the gravitational component along the incline *mg·sinα* significantly increases and emerges as the dominant factor driving downslope straw migration. Concurrently, the centrifugal force component *mω²r·cosγ* increases with increasing rotational speed, further exacerbating the lateral dispersion of the straw.

Moreover, the bending angle *θ* governs the directional trajectory of the straw during detachment: A smaller *θ* facilitates lateral straw dispersal, whereas a larger *θ* results in a more horizontally oriented backward trajectory of straw movement. the coefficient of friction between the straw and the tangential plane of the rotary blade affects the magnitude of the frictional force and thus influences the initial speed and direction of the movement of the straw.

The kinetic energy obtained by the straw during the impact of the rotary tillage blade can be expressed, as shown in Equation 3.

(3) $$E_{k}\;=\;\frac{1}{2}mv_{s}^{2}$$

In the formula: *m* represents the mass of the straw (kg);
*v_s_* is the initial velocity of the straw when it detaches from the blade (m/s).

The value of *v_s_* is mainly determined by the linear velocity *v_t_* of the rototiller, which is given by *v_t_ = ωR*, and the relative motion between the straw and the blade surface. The forward farming speed *v* affects the contact angle between the straw and the blade surface as well as the rotation angle of the blade axis at the moment of detachment. When moving at a low speed, the straw is more likely to be ejected when the rotation angle of the blade shaft approaches its maximum value (that is, the direction of the tip line velocity is close to being horizontally backward), thereby obtaining a more similar initial velocity to the tip line velocity of the blade, increasing the value of *v_s_* and the displacement.

Under the condition that the angular velocity *ω* (*ω= 2πn/60*) of the blade shaft is fixed, the forward farming speed *v* determines the size of the soil-cutting pitch *s*. Let the number of blades in the rotary tillage blade assembly be *z*, then the soil-cutting pitch is shown in Equation 4.

(4) $$s=\frac{2\pi v}{z\omega }$$

A smaller soil-cutting pitch results in a higher frequency of stubble disturbance by consecutive blades per unit length. As the velocity *v* decreases, the displacement *s* correspondingly decreases, resulting in a significant increase in the probability that a single straw is sequentially struck and scattered by multiple blades during the operational stroke. Consequently, the cumulative displacement of the straw markedly increases with decreasing velocity.

In summary, the force and movement behavior of straw in sloping rotary tillage operations are jointly influenced by the rotational speed of the blade shaft, the speed of the rotary tillage operation, the structure of the tangent blade, the slope angle, and the frictional characteristics. Under the coupled action of the component force of gravity and the centrifugal force, straw is prone to asymmetric migration toward the lower side of the slope. This analysis provides a theoretical basis for the subsequent experimental research and parameter optimization.

### 2.3 Test conditions

**Figure 5:**
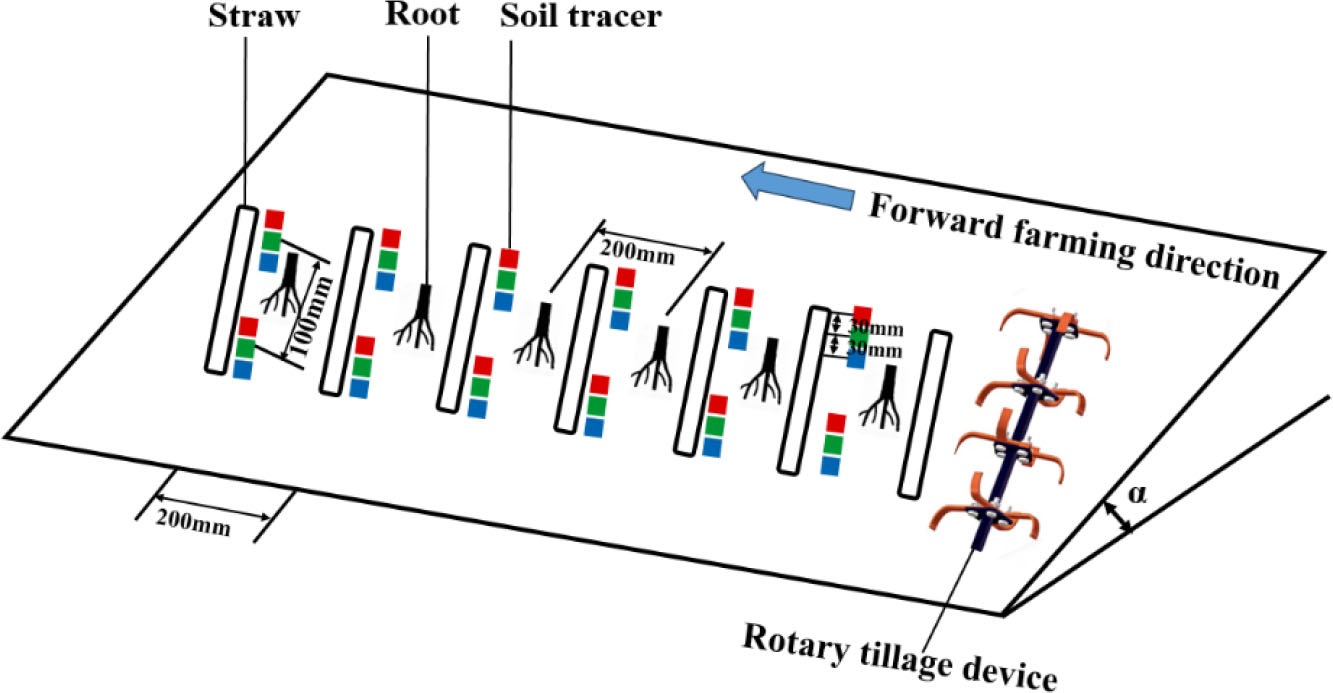
Burial layout scheme of straw-root-soil complex.

The experiment was conducted with the aim of replicating the actual scenario of returning corn straw to a field after it was harvested on sloping land. The objective was to determine the migration laws of the straw–root–soil complex after rotary tillage operations on sloping land. Specifically, yellow loam soil from the North China was utilized in the experiment. The collected corn straw (with a moisture content of 20%) was processed to match the typical length after straw incorporation. Straw segments measuring approximately 200 ± 10 mm in length and 20 ± 10 mm in diameter were selected as test samples. To observe straw displacement following rotary tillage operations, the straw segments were sequentially numbered and arranged parallel to the rotary blade shaft. Samples of corn stubble at the harvest stage were taken, and the soil was removed. As much of the root systems, such as the main roots and lateral roots, was retained as possible (the length of the remaining straw was 50 mm ±10 mm, and the moisture content was 30% ± 3%). In accordance with the measured data, such as the plant spacing of corn in the field, simulated burial was carried out in the sloping soil trough. Soil displacement was measured using the tracer method (Sun et al., 2022c). Aluminum blocks with a density similar to that of soil (dimensions: 10 mm × 10 mm × 10 mm) were selected as tracers (Zeng et al., 2020). Each aluminum block was assigned a unique identification number and embedded at depths of 30 mm, 60 mm, or 90 mm below the surface. The burial configuration is illustrated in **Figure 5**.

Prior to the experiment, the experimental soil (with an initial moisture content of approximately 15% ± 2%) was laid and compacted in the soil bin. The soil bin platform was then adjusted to the target slopes (5°, 10°, and 15°) via a hydraulic cylinder. The rotary tillage mechanism was subsequently activated to commence tillage operations along the leveled contour slope. A designated operational area measuring 2000 mm × 1200 mm at the center of the soil trough platform was selected as the stable velocity work zone. At each tracer measurement point, the initial coordinates (*X*_0_, *Y*_0_) of all the complex components (straw, roots, and soil clods) were recorded in the coordinate measuring frame, and measurements were acquired across three vertical layers: top, middle, and bottom.

### 2.4 Test plan

**Figure 6:**
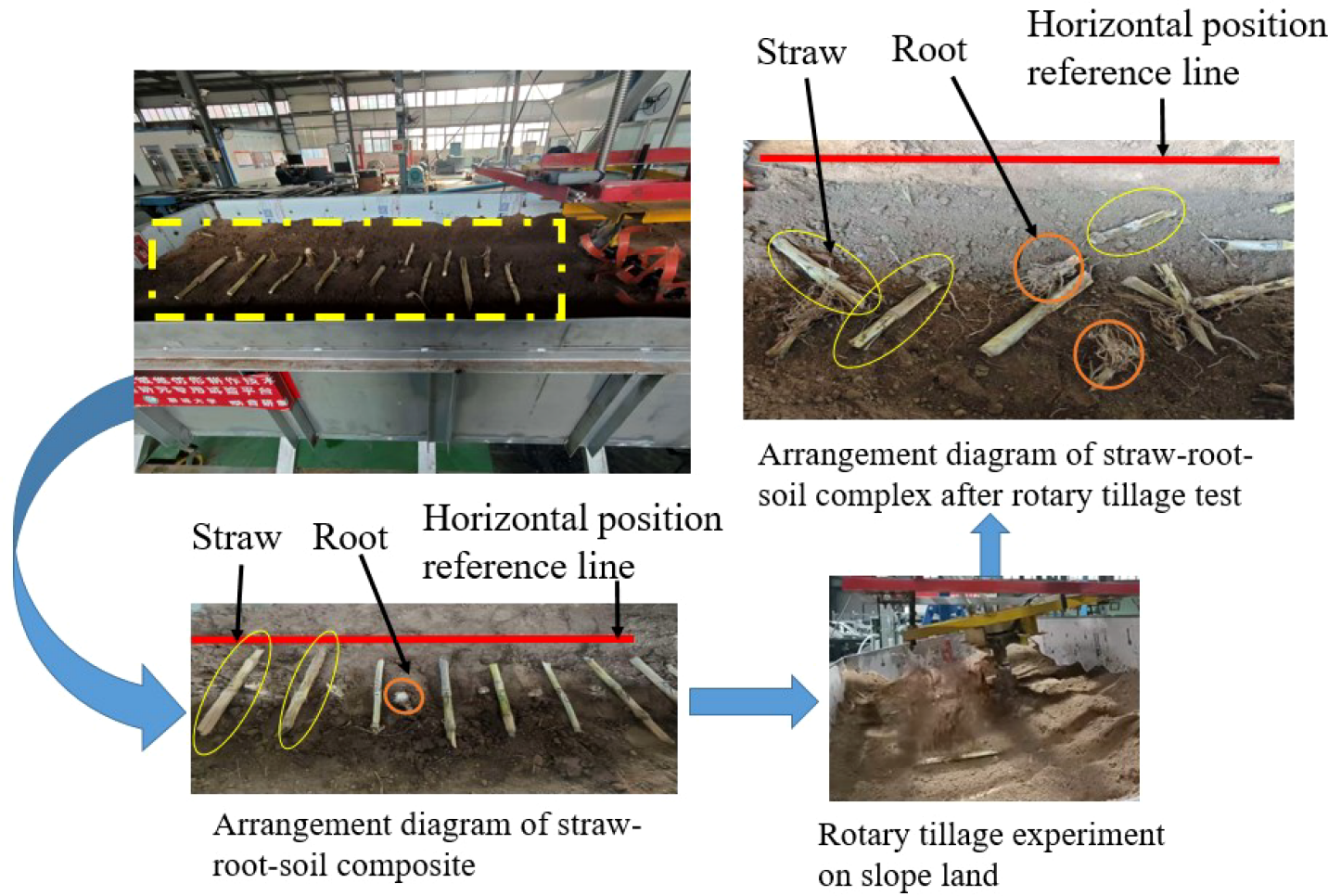
Experimental procedure of sloping field rotary tillage test.

To investigate the influence of slope conditions (slope angle) and machine operating parameters (blade shaft rotational speed, forward farming speed) on the transport characteristics of the straw-root-soil complex, single-factor experiments were conducted. The experimental design employed a control variables approach based on three key factors: slope angle (*α*), blade shaft rotational speed (*n*), and forward farming speed (*v*). According to field investigations conducted in the North China region, the typical tillage depth of micro-tillers is predominantly within the range of 8–10 cm. In this study, the tillage depth was standardized to 10 cm. Based on preliminary field investigations of sloping farmland in hilly and mountainous areas, it was found that the slope angles of common tillage operations are mainly concentrated in the range of 5°–15°. Tillage operations within this slope range are highly prone to tillage erosion (i.e., soil migration from the upslope side to the downslope side). A slope of 15° is considered a steep slope. Beyond this gradient, the operational safety and stability of agricultural machinery cannot be guaranteed, and steep slopes in hilly and mountainous areas are mainly used for forestry development. The slope gradient was established at three levels: 5°, 10°, and 15°; the blade shaft rotational speed was set to 200 r/min, 250 r/min, and 300 r/min; and the forward farming speed was configured at 0.2, 0.4, 0.6, 0.8, and 1.0 km/h. All tests were repeated in triplicate, and the results are presented as mean values. During the experimental process, a coordinate measurement system was employed to record the positions of each component of the complex (straw, roots, and soil clods) before and after the operation. The horizontal displacement *ΔX* (defined as positive in the direction opposite to the operation-al direction) and lateral displacement *ΔY* (defined as positive in the downhill direction) were calculated accordingly. The experimental workflow is illustrated in **Figure 6**.

### 2.5 Analysis of straw migration based on high-speed imaging

**Figure 7:**
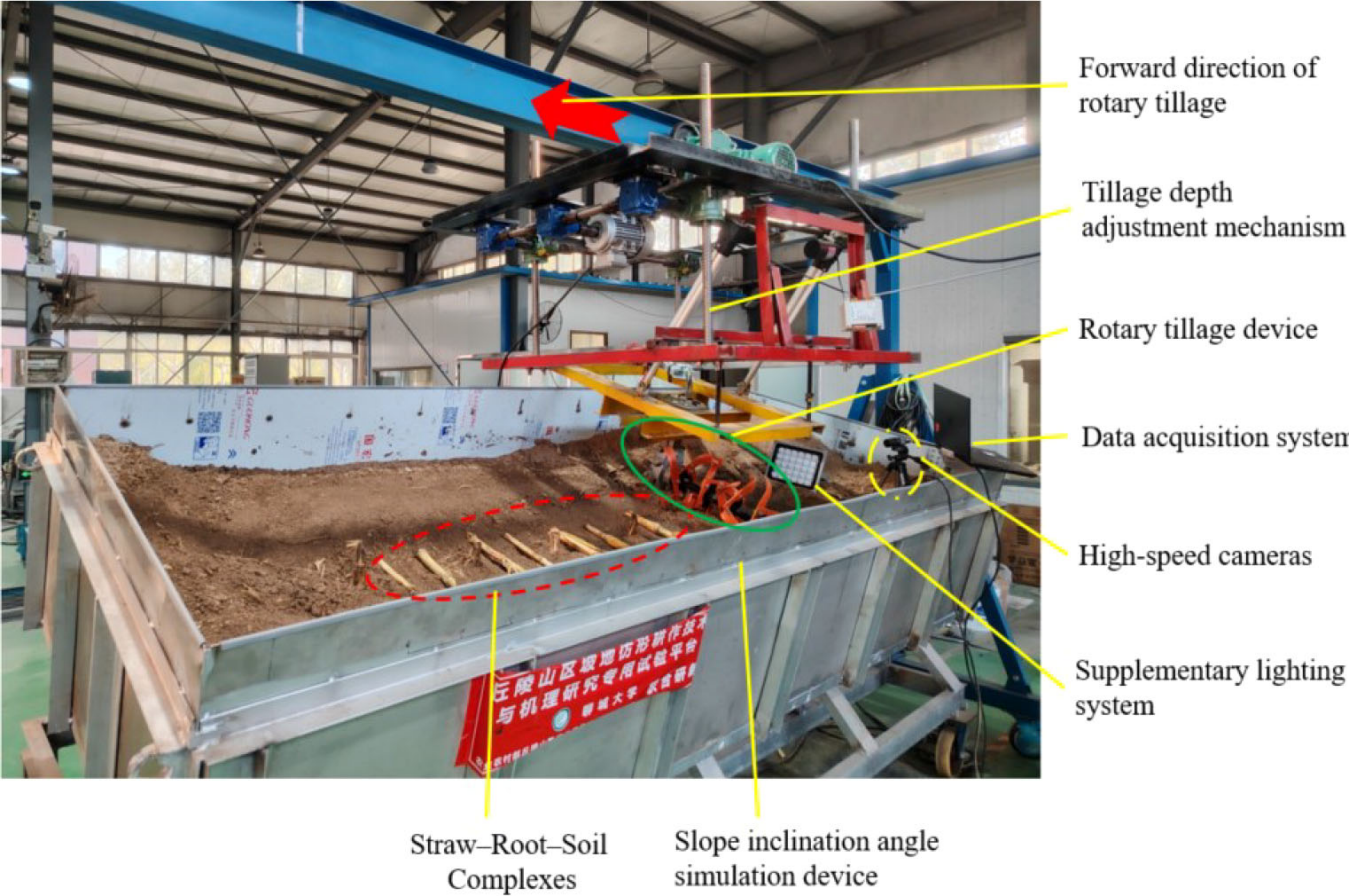
Bench test diagram based on high-speed imaging technology.

To further verify the instantaneous movement characteristics of straw under the action of rotary blades and to provide an intuitive dynamic basis for subsequent quantitative analysis, this study introduced high-speed imaging technology to dynamically capture the ejection process of the complex. The experiment employed a high-speed camera equipped with a Variable-focus lens and maximum frame rate of 815 frames per second. The camera was positioned behind the soil bin platform, with its lens axis perpendicular to the rotary blade shaft, to clearly record the entire process of straw from being struck by the blade to being ejected. The bench test diagram based on high-speed imaging technology is shown in **Figure 7**.

**Figure 8:**
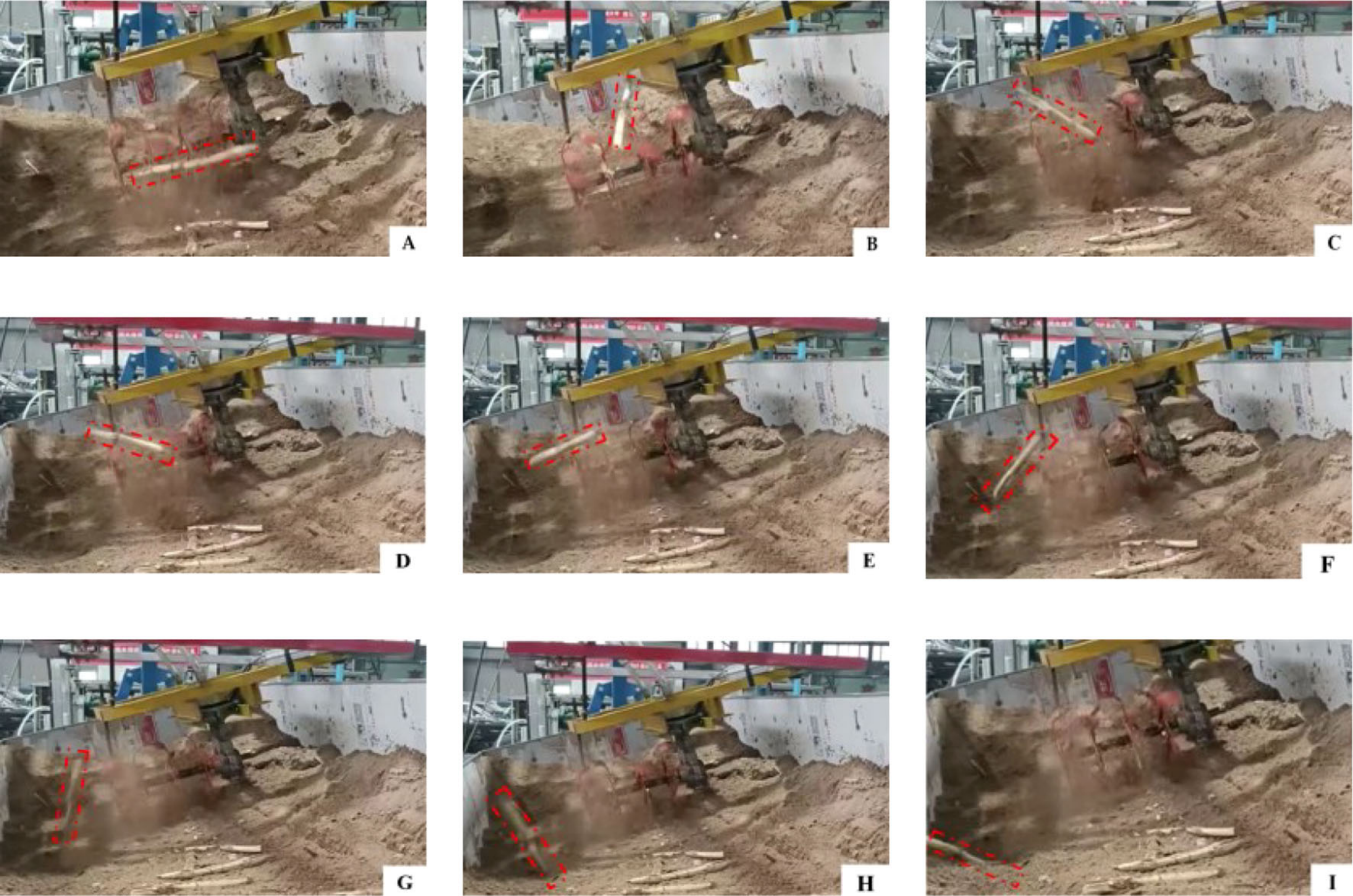
Straw movement trajectory captured by high-speed camera technology. The movement trajectory of the straw is represented by Figures **A** to **I**, which show sequential frames of the progressive movement of straw (marked by red dashed lines) under a slope gradient of 15°, blade shaft rotational speed of 300 r/min, and forward farming speed of 0.6 km/h. The red dotted lines in each frame represent the instantaneous posture of the representative straw.

The image sequences captured by high-speed imaging were imported into Kinovea 2024 software for analysis. The main analysis contents include: the spatial trajectory of straw (marked with red dashed lines in the images). Under the operating conditions of a slope gradient of 15°, a blade shaft rotational speed of 300 r/min, and a forward farming speed of 0.6 km/h, the straw trajectory captured by high-speed imaging (as shown by the red dashed line in **Figure 8**) exhibits the following typical characteristics. The overall trajectory exhibits a distinctly asymmetric curved shape. After being struck by the blade, the straw is not ejected straight horizontally; instead, it rapidly deflects downslope, forming a parabolic trajectory that bulges toward the lower side of the slope. In the initial stage of the trajectory (within approximately 0–0.05 m after detachment from the blade), the direction is primarily backward; then it quickly bends downslope, and the endpoint position significantly deviates from directly behind the blade, with a large lateral displacement toward the lower side of the slope.

**Figure 9:**
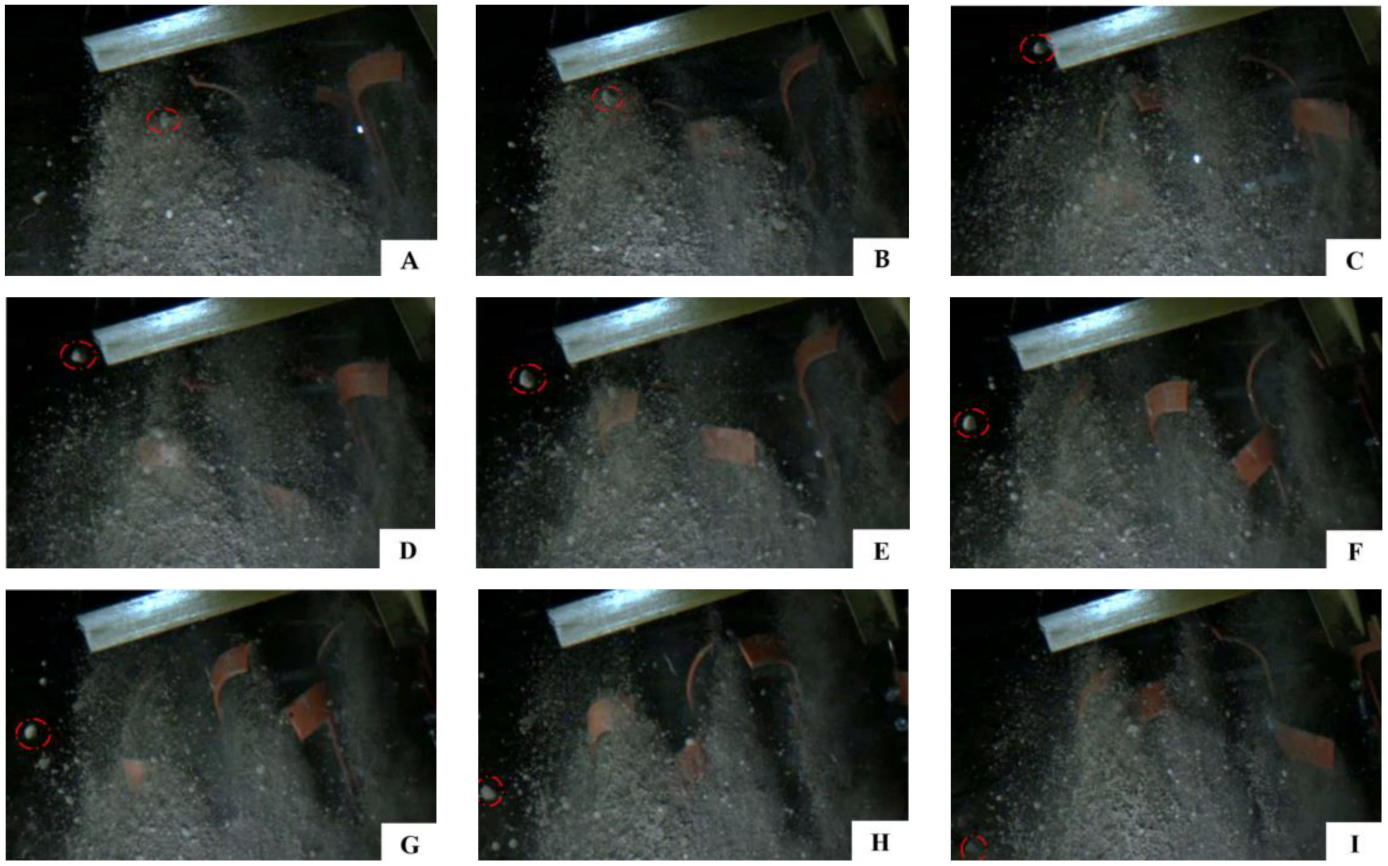
The movement trajectory of the soil blocks is represented by Figures **A** to **I**, captured by high-speed imaging technology. The diagram shows the displacement pattern of a typical soil block (marked with a red dotted line) under the conditions of a slope gradient of 15°, blade shaft rotational speed of 300 r/min, and forward farming speed of 0.6 km/h.

The soil migration trajectory captured by high-speed imaging technology is shown in **Figure 9**. In the figure, red dashed lines are used to mark the movement path of a typical soil clod. In terms of trajectory morphology, after being struck and detached by the rotary blade, the soil clod exhibits a distinctly parabolic trajectory that bulges downward along the slope.

## 3 Results and discussion

### 3.1 Influences of farming parameters on the migration of complex systems

#### 3.1.1 Impact of the forward farming speed

**Figure 10:**
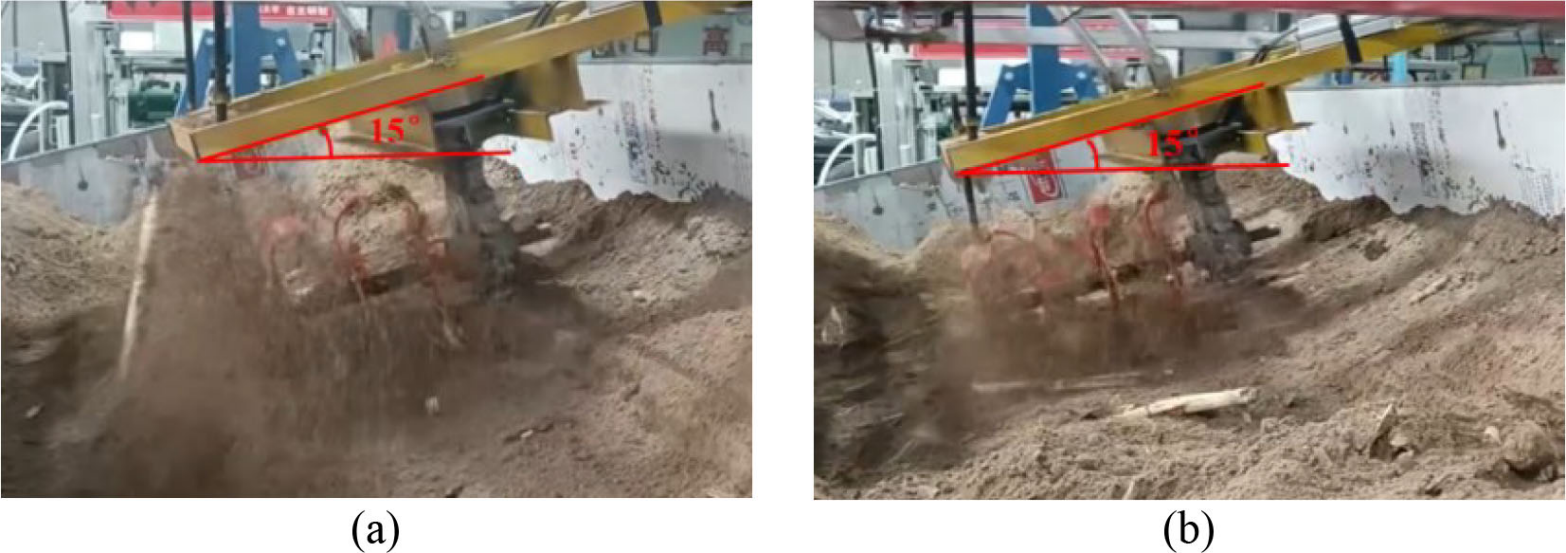
Experimental photographs showing the effect of forward farming speed on the average displacement of the straw–root–soil complex. **(a)** The test image under the operating condition of 0.6 km/h; **(b)** The test image under the operating condition of 1.0 km/h.

**Figure 11:**
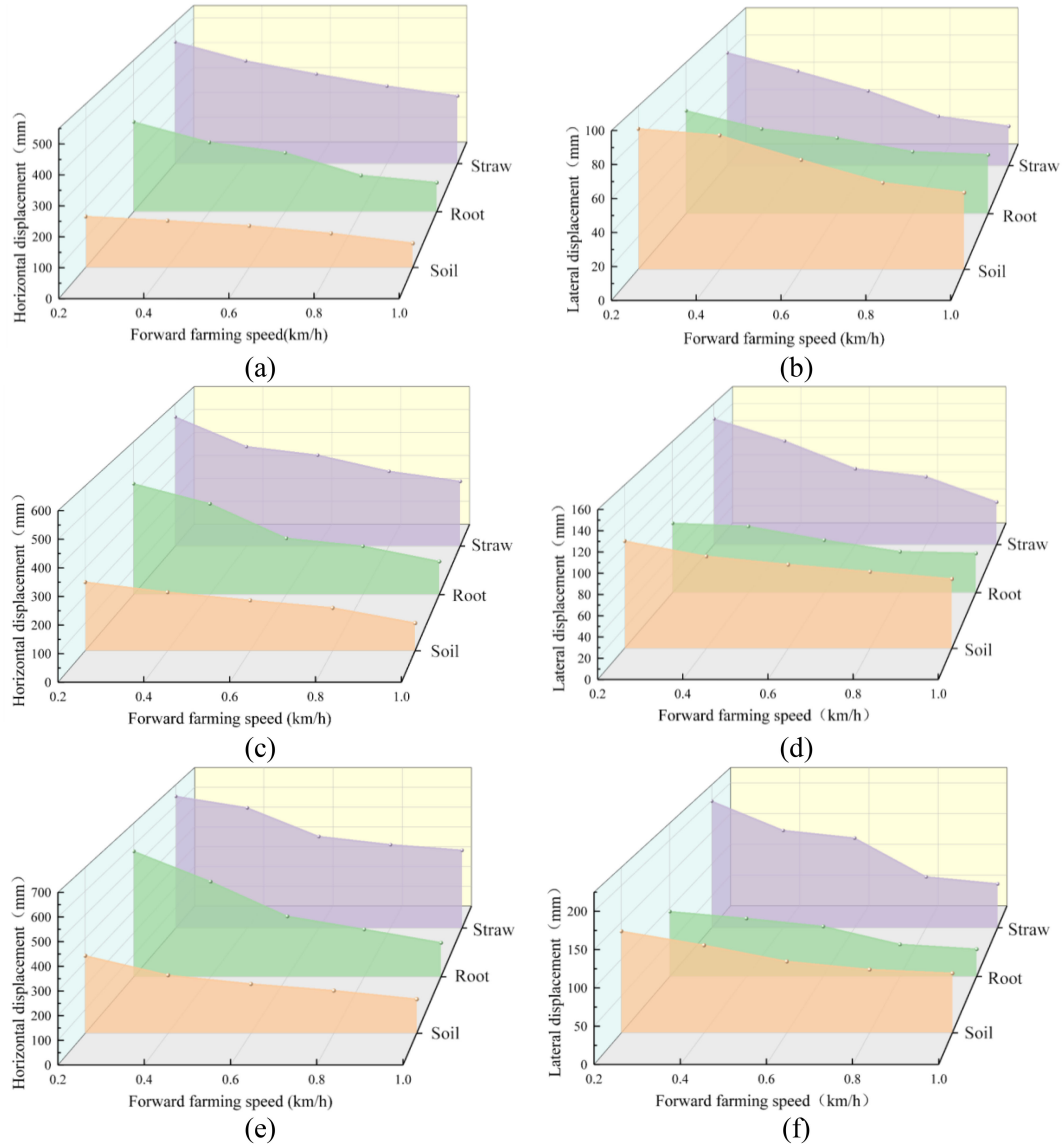
Effect of forward farming speed on the average displacement of the straw–root–soil complex. **(a)** Horizontal displacement at 5° slope; **(b)** Lateral displacement at 5° slope; **(c)** Horizontal displacement at 10° slope; **(d)** Lateral displacement at 10° slope; **(e)** Horizontal displacement at 15° slope; **(f)** Lateral displacement at 15° slope.

Under fixed slope gradient (5°, 10°, and 15°), blade shaft rotational speed (300 r/min), and tillage depth (100 mm) conditions, the effect of the forward farming speed on the average displacement of the soil–tool complex was investigated. A schematic diagram of the experimental process is shown in **Figure 10** (15-degree slope angle, 300r/min), and the influence of the forward farming speed on the displacement of the complex is illustrated in **Figure 11**. As the velocity increases from 0.2km/h to 1.0 km/h, the horizontal and lateral displacements of the straw, stubble, and soil significantly decrease. In particular, at a slope of 15°, as the velocity increases from 0.2km/h to 1.0 km/h, the horizontal displacement of the straw decreases from 644mm to 380mm, whereas its lateral displacement decreases from 199 mm to 69 mm. The horizontal dis-placement of the root stubble decreased from 571 mm to 150 mm, whereas the lateral displacement decreased from 95mm to 40mm. Straw displacement is most significantly influenced by the velocity, particularly within the low-speed range (0.2–0.6 km/h), with both the horizontal and lateral displacements decreasing by more than 40%. The displacement of the root stubble is similar to that of the soil, albeit with a smaller magnitude of variation. The results demonstrate that the forward farming speed has the most significant influence on the motion of lightweight, high-porosity straw particles and thus serves as a critical parameter for suppressing the overall migration of the complex system. When operating on a 15° slope, as the speed increases from 0.2km/h to 1.0km/h, the horizontal displacement of the straw decreases by approximately 40.9%, and the lateral displacement is reduced by approximately 65.3%. Among the three components, straw has the largest contact area and the highest contact frequency with the rotary blade shaft. A lower forward farming speed allows more sufficient interaction between the straw and the rotary blade shaft, making it more sensitive to changes in the impact frequency of the rotary blades. Furthermore, the flexibility of straw enables it to be repeatedly struck by multiple rotary blades at a low forward farming speed, thus providing the possibility for straw to accumulate greater displacement. The underlying mechanism is that a lower forward farming speed prolongs the duration of the interaction between the rotary blade and the soil complex per unit area, resulting in an increase in the number of disturbances and the energy input. Consequently, the soil complex is more thoroughly dispersed under these conditions, increasing the displacement. Compared with the lateral displacement, the horizontal displacement is more sensitive to variations in the rotary tillage speed, further supporting that the forward farming speed serves as a critical parameter for controlling the projection distance of the soil complex. Previous studies have shown that lower forward operating speed during mouldboard plough tillage results in greater soil and straw displacement (Eltom et al., 2015), which is consistent with the pattern observed under the continuous cutting mechanism of rotary tillage (Eltom et al., 2015).

#### 3.1.2 Impact of the blade shaft rotational speed

**Figure 12:**
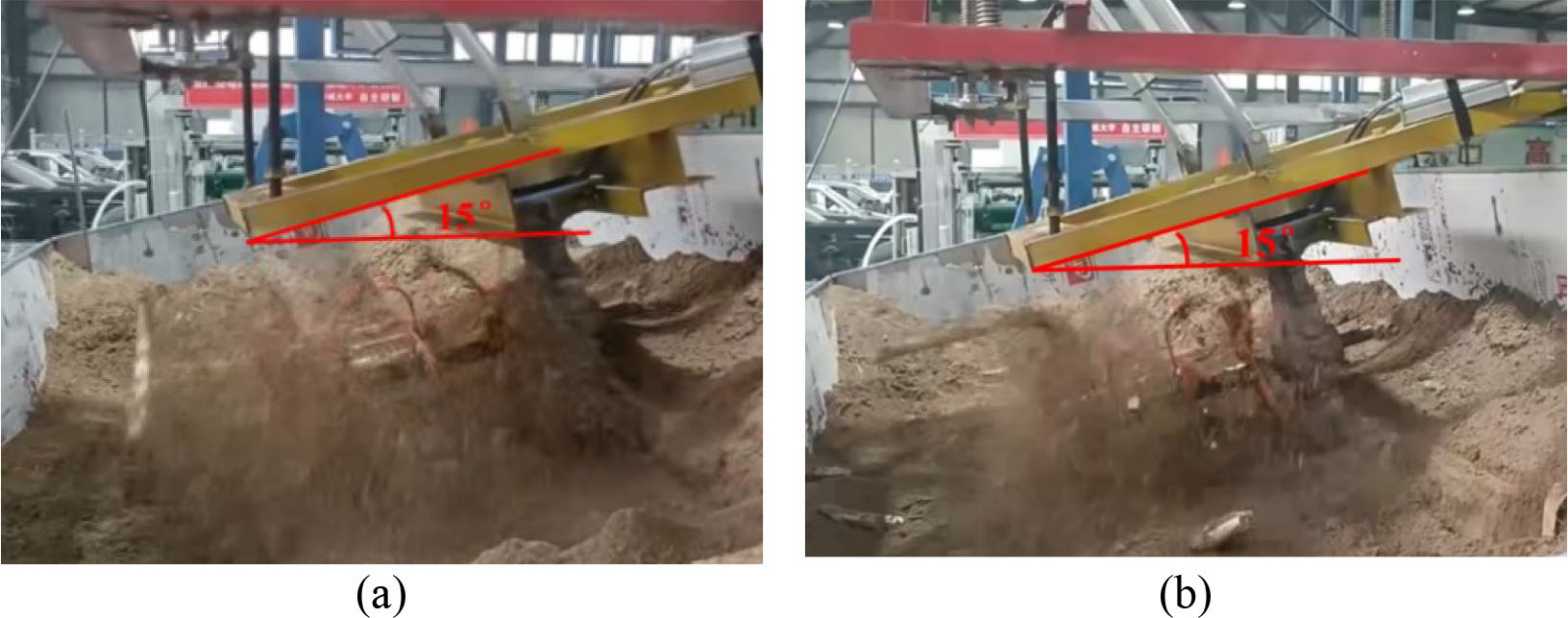
Experimental photographs showing the effect of blade shaft rotational speed on the average displacement of the straw–root–soil complex. **(a)** The test image under the operating condition of 300r/min; **(b)** The test image under the operating condition of 200r/min.

**Figure 13:**
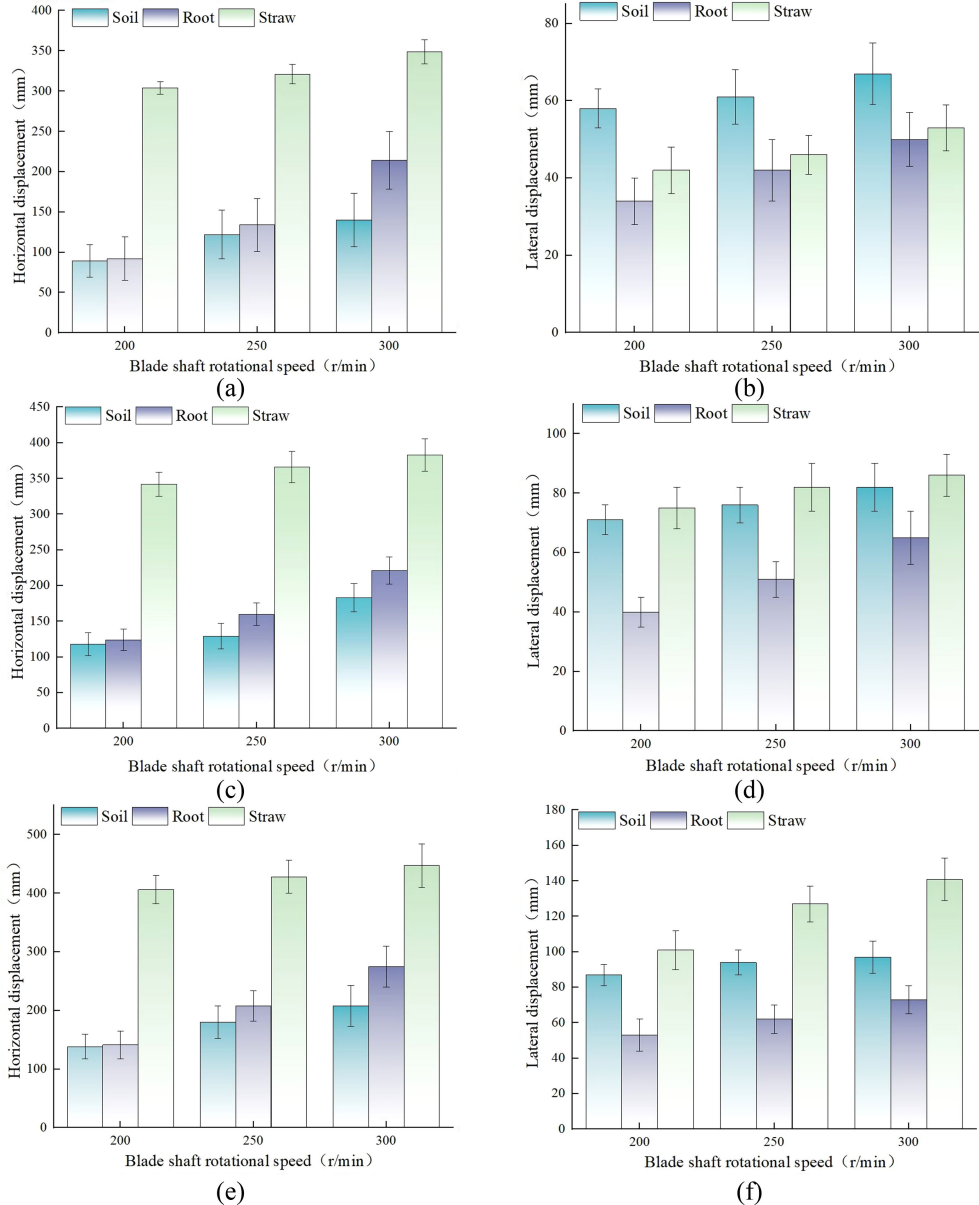
Effect of blade shaft rotational speed on the average displacement of the straw–root–soil complex. **(a)** Horizontal displacement at 5° slope; **(b)** Lateral displacement at 5° slope; **(c)** Horizontal displacement at 10° slope; **(d)** Lateral displacement at 10° slope; **(e)** Horizontal displacement at 15° slope; **(f)** Lateral displacement at 15° slope.

At a fixed slope angle (5°, 10°, and 15°), forward farming speed (0.6 km/h), and tillage depth (100 mm), the influence of the rotational speed of the blade shaft (200, 250, and 300 r/min) on the displacement of the straw–root–soil complex was investigated. A schematic diagram of the experimental process is shown in **Figure 12** (15°,0.6 km/h), and the influence of the blade shaft rotational speed on the displacement of the complex is illustrated in **Figure 13**. On steep downhill slopes, root stubble is more sensitive to greater rotational speeds, and its horizontal displacement surpasses that of straw and soil. This finding indicates that following structural damage, root stubble becomes more prone to high-speed scattering. The inherent configuration of the root stubble increases the probability of contact with the rotary blades. Additionally, the lateral displacement of the straw notably increases with increasing rotational speed, particularly at a rotational speed of 300 r/min. This finding indicates that under high-speed conditions, the coupled effects of the centrifugal force and the gravitational component along the slope intensify, leading to a significantly elevated risk of lateral drift. Compared with that under gentle slope conditions (e.g., 5°), the complex lateral displacement increases more with increasing blade shaft rotational speed under steeper slope conditions (15°), indicating that the slope angle significantly amplifies the driving effect of the rotational speed on the lateral migration of the material. This phenomenon is directly attributable to the increased gravitational component acting parallel to the inclined surface. Therefore, in operations on steep slopes, controlling the blade shaft rotational speed is crucial to mitigate excessive lateral dispersion of residues, particularly straw and stubble. Zhao et al. (2025) reported that higher blade shaft rotational speed increased soil fragmentation and straw mulching coverage, which is essentially driven by the greater kinetic energy, larger displacement and more intense motion of soil and straw particles under high rotational speeds. The positive correlation between blade shaft rotational speed and displacement demonstrated by other scholars is highly consistent with the findings of this chapter (Xu et al., 2022d; Fang et al., 2016).

Specifically, after being struck by the blade, the initial velocity of the straw is approximately equal to the tangential velocity of the blade tip. According to Newton’s first law of motion (inertia), the straw tends to maintain this velocity direction (tangential direction), resulting in lateral scattering.

Meanwhile, the rotating blade imparts centrifugal force to the straw, as shown in Equation 5.

(5) $$\;F_{c}=m\omega^{2}r$$

In the equation, *m* represents the mass of the straw(kg);
*ω* denotes the angular velocity(rad/s);
*r* signifies the instantaneous radius of rotation(mm).

An increase in the rotational speed *n* results in a quadratic increase in the centrifugal force, as *F_c_ ∝ n²*, which drives the straw to move radially outward. This motion, combined with the tangential direction of the blades, results in a lateral parabolic trajectory. The interaction forces between the soil and straw weaken, whereas the blade-induced “ejection effect” on the soil becomes more pronounced at higher rotational speeds. As a result, soil particles and straw are projected laterally, decreasing the soil-induced drag on the straw and thereby increasing its lateral displacement.

#### 3.1.3 Influence of the slope angle

**Figure 14:**
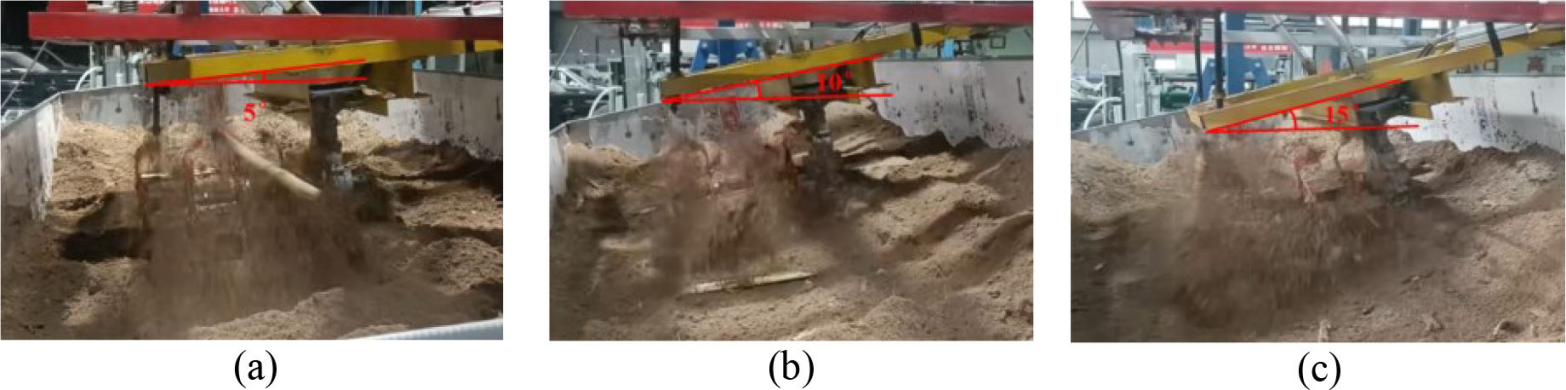
Experimental photographs showing the effect of slope gradient on the average displacement of the straw–root–soil complex. **(a)** The influence of a 5° slope on the displacement of the complex structure; **(b)** The influence of a 10° slope on the displacement of the complex structure; **(c)** The influence of a 15° slope on the displacement of the complex structure.

**Figure 15:**
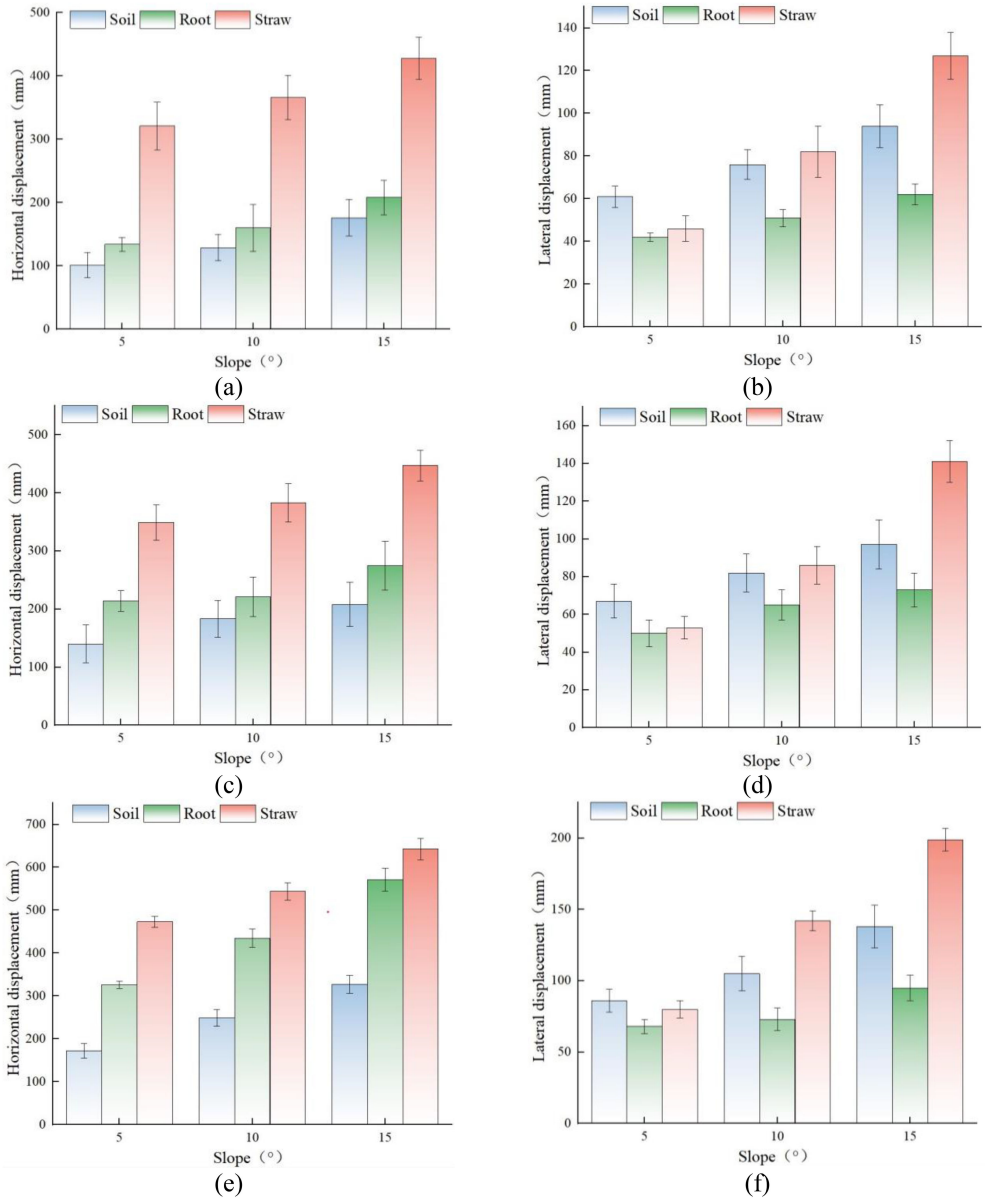
Effect of slope gradient on the displacement of each component of the straw–root–soil complex at different depths. **(a)** Horizontal displacement at 0.6 km/h, 250 r/min; **(b)** Lateral displacement at 0.6 km/h, 250 r/min; **(c)** Horizontal displacement at 0.6 km/h, 300 r/min; **(d)** Lateral displacement at 0.6 km/h, 300 r/min; **(e)** Horizontal displacement at 0.2 km/h, 300 r/min; **(f)** Lateral displacement at 0.2 km/h, 300 r/min.

Under the predetermined operational parameters of a fixed blade shaft rotational speed (300 r/min), forward farming speed (0.6 km/h), and tillage depth (100 mm), the influence of the slope angle (5°, 10°, and 15°) on the displacement of the complex unit was investigated. The experimental procedure is illustrated in **Figure 14**. The experimental results (**Figure 15**) demonstrate that slope angle is the predominant natural factor driving the lateral translocation of the complex materials, particularly straw residues, within the studied terrain. When the slope increased from 5° to 15°, the horizontal displacement of the straw increased from 349 mm to 447 mm (an increase of approximately 28.1%), and the lateral displacement increased from 53 mm to 141 mm (an increase of approximately 166.0%). The horizontal displacement of the root stubble increased from 214 mm to 275 mm (an increase of approximately 28.5%), and the lateral displacement increased from 50 mm to 73 mm (an increase of approximately 46.0%). The horizontal soil displacement increased from 140 mm to 208 mm, representing an increase of approximately 48.6%. The lateral displacement increased from 67 mm to 97 mm, corresponding to a growth of approximately 44.8%. The influence of slope on the lateral displacement of straw is the most prominent (with an increase of up to 166%), far exceeding that of stubble and soil. In sloping land operations, the susceptibility of straw to asymmetric migration along the downslope direction is increased because of the significant influence of gravitational components. Owing to the cohesive forces between soil particles, the displacement response exhibits a relatively gradual trend. Under identical operational parameters (e.g., *v* = 0.6 km/h and *n* = 300 r/min), as the slope angle increases from 5° to 15°, the average lateral displacement of the straw increases by approximately 166%, whereas that of the stubble and soil exhibit similar trends; in contrast, the horizontal displacement of the components is less influenced by changes in the slope. This result clearly demonstrates that during hillslope rotary tillage operations, the gravitational component along the slope is the fundamental cause of the asymmetric translocation of straw and soil in the downslope direction. Zhao et al. (2016) pointed out that slope gradient is a key factor influencing tillage displacement, and there is a significant positive correlation between the two, which is in excellent agreement with the conclusions drawn in this chapter.

During hillslope rotary tillage operations, the lateral transport of crop residue, root stubble, and soil complexes is predominantly driven by the gravitational force component parallel to the slope. When the slope surface has an inclination angle *α*, assuming that the mass of the straw complex is *m*, the cutting speed of the rotary blade is *Vc*, and the coefficient of friction between the straw and soil is *μ*, the force equilibrium equation along the slope direction can be expressed as follows.

In the Equation 6:

(6) $$ma=mg\sin\alpha -\mu mgcos\alpha +F_{cut}cos\psi$$

*a* is acceleration of the complex body along the slope surface(m/s^2^);
*F_cut_* is cutting force of the rotary blade(N);
*ψ* is angle between the cutting force and the slope surface(°).

Owing to the flexible deformation characteristics of straw, equivalent stiffness coefficient *k* and damping coefficient *c* of flexible materials are incorporated. By integrating these parameters with the governing equations of motion, a coupled dynamic model is formulated, as shown in Equation 7.

(7) $$m\;x^{\prime \prime }+cx^{\prime }+kx=mgsin\alpha -\mu mgcos\alpha +F_{cut}\left(\omega t\right)\cos\psi$$

In this equation, *F_cut(ωt)_* represents the time-dependent cutting force characterized by periodic variation over time. *x* is the displacement of the complex along the slope direction (m), *x*′ is the velocity (m/s), and *x*″ is the acceleration (m/s^2^).

As the slope angle *α* increases from 5° to 15°, the component of the gravitational force parallel to the slope surface, *mg·sinα*, increases by approximately 196%. This is much greater than the change in the frictional resistance term, *μmgcosα*, which decreases by only approximately 8.7%. Owing to this mechanical characteristic, the lateral displacement of the straw increases by 166%, which is significantly greater than that of the root stubble and soil. Consequently, an increase in the slope angle leads to a substantial increase in the gravitational component parallel to the slope surface, which is the fundamental cause underlying the asymmetric downslope migration of the complex material.

### 3.2 Mechanism of the impact of straw content on soil displacement

The quantity of straw incorporated is a critical factor influencing the quality of rotary tillage operations and the erosion resistance of the soil (Xia et al., 2026). Through bench-scale experiments, the influence of different straw mulching rates (0.4, 0.8, and 1.2 kg/m²) on soil displacement was systematically investigated. Furthermore, by accounting for the interactive effects of the slope gradient and operational speed, the regulatory mechanism of straw mulch on soil stability on sloping lands was elucidated.

The experiment was conducted on the slope rotary tillage test platform using soil with properties identical to that used in the prior tests. Three levels of straw content were established: 0.4 kg/m² (low coverage), 0.8 kg/m² (medium coverage), and 1.2 kg/m² (high coverage). The straw, with a uniform length of 200 mm, was randomly dispersed over the soil surface. Experimental slope gradients of 5°, 10°, and 15° were established, with a forward farming speed of 0.6 km/h, a blade shaft rotational speed of 300 r/min, and a tillage depth of 100 mm. Each experimental group was subjected to three repeated trials, and soil displacement was quantified using the aluminum tracer block method.

#### 3.2.1 Influence law of straw content on soil horizontal displacement

**Figure 16:**
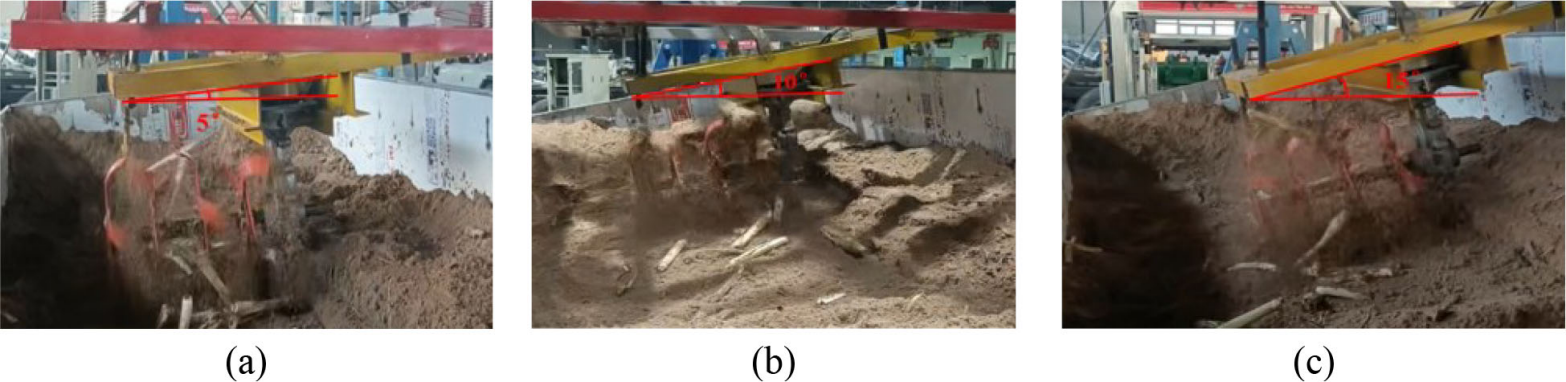
Experimental photographs showing the effect of different straw contents on soil horizontal displacement. **(a)** is the test image under the operating condition of 5°; **(b)** is the test image under the operating condition of 10°; **(c)** is the test image under the operating condition of 15°.

**Figure 17:**
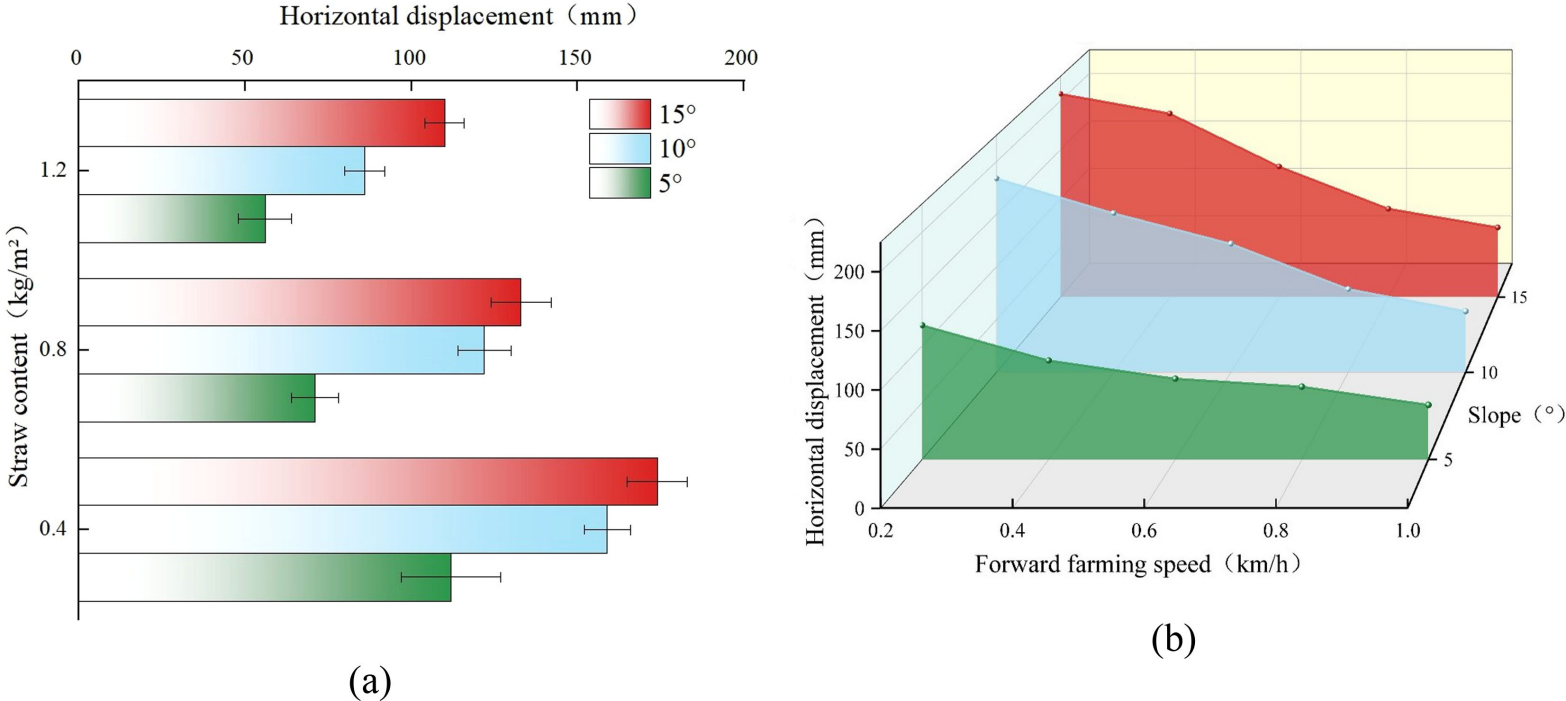
Effect of straw content on the variation law of soil horizontal displacement. **(a)** The effect of straw content on soil horizontal displacement under different slope gradients; **(b)** The effect of forward farming speed on soil horizontal displacement under different slope gradients.

As shown in **Figure 16** (0.6 km/h, 0.4 kg/m² operating condition test chart), the effects of varying straw contents on soil displacement were investigated. As illustrated in **Figure 17a**, at a forward farming speed of 0.6 km/h, a blade shaft rotational speed of 300 r/min, and a tillage depth of 10 cm, the horizontal soil displacement was clearly inversely correlated with increasing straw content across different slope gradients. Specifically, as the straw content increases, the horizontal soil displacement gradually decreases on terrains with slopes of 5°, 10°, and 15°. **Figure 17b** illustrates the relationship between forward farming speed and soil displacement across different slope gradients under the following conditions: straw content of 0.8 kg/m², blade shaft rotational speed of 300 r/min, and tillage depth of 10 cm. Under a slope of 5°, an increase in straw coverage from 0.4 kg/m² to 1.2 kg/m² resulted in a reduction of soil horizontal displacement from 112 mm to 56 mm, representing a 50% decrease. At a gradient of 10°, the corresponding displacement decreased from 159 mm to 86 mm, representing a reduction of 45.9%; whereas at a 15° gradient, the displacement declined from 174 mm to 110 mm, corresponding to a 36.8% decrease. With increasing straw content, the horizontal displacement of the soil significantly decreased, indicating that straw mulching effectively inhibited the backward scattering of soil. Under gentle slope conditions (5–10°), straw incorporation has a more pronounced inhibitory effect on soil displacement. On steep slopes (15°), although the absolute displacement of the soil decreases with increasing straw content, the magnitude of this decrease is reduced, which may be attributable to the increased gravitational downslope component.

The underlying reason is that on sloped terrain, the gravitational force acting on a soil particle can be resolved into two components: a normal force perpendicular to the slope surface (*mg·cosα*) and a tangential force directed downslope along the slope surface (*mg·sinα*). As the slope angle (*α*) increases, the tangential component (i.e., the gravitational force component) significantly increases, which results in the soil particles accelerating more readily downslope when they are disturbed, thereby increasing the horizontal displacement of the soil. Under gentle slope conditions (5–10°), the gravitational force components are relatively minor, and the primary driving force for soil displacement is the mechanical operation of the equipment. The resistance provided by straw mulching is sufficient to effectively counteract the corresponding driving force component, resulting in a significant suppression effect and a marked reduction in the soil displacement. On steep slopes (15°), the gravitational component is significantly increased, and this force serves as a critical additional force that drives soil movement downslope. The restraining force associated with the increase in the straw content may be insufficient to fully counteract the amplified gravitational component, resulting in persistently high absolute soil displacement and a relatively diminished reduction in the soil displacement associated with the increase in straw con-tent. Therefore, when soil conservation measures are formulated, the slope gradient must be considered, and additional soil and water conservation techniques may need to be combined in areas with steep slopes to increase their effectiveness (Chen et al., 2022a).

#### 3.2.2 Influence law of the straw content on soil lateral displacement

**Figure 18:**
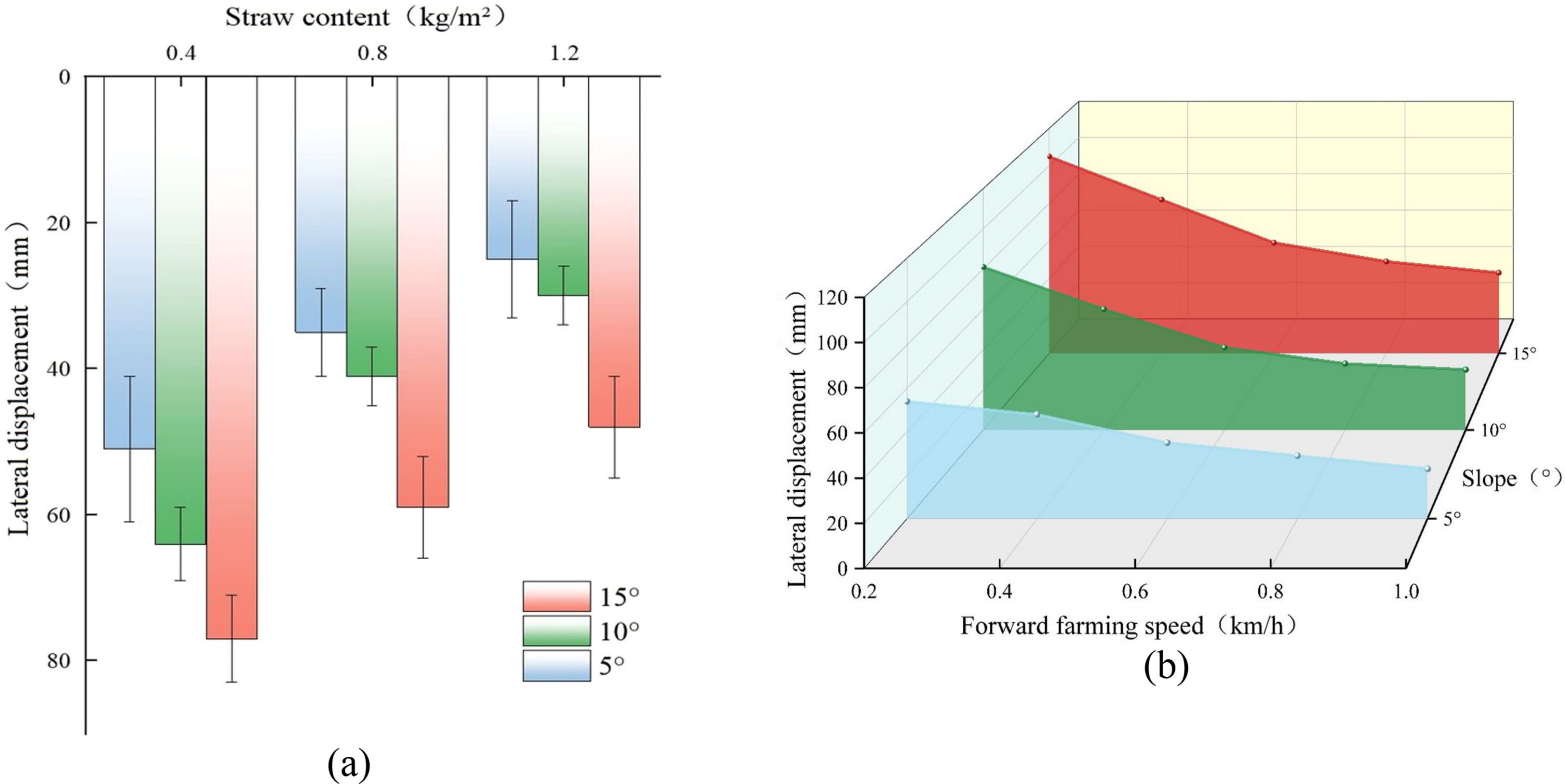
Effect of straw content on the variation law of soil lateral displacement. **(a)** The effect of straw content on soil lateral displacement under different slope gradients; **(b)** The effect of forward farming speed on soil lateral displacement under different slope gradients.

As illustrated in **Figure 18a**, under a forward farming speed of 0.6 km/h, a blade shaft rotational speed of 300 r/min, and a tillage depth of 10 cm, the relationship be-tween the straw content and slope in terms of the lateral displacement of the soil was investigated. An increase in the straw content effectively suppresses lateral soil dis-placement, and a higher straw content increases the erosion resistance of the soil. The relationships between the forward farming speed and lateral soil displacement under varying slopes, with a straw content of 0.8 kg/m², a blade shaft rotational speed of 300 r/min, and a tillage depth of 10 cm, are shown in **Figure 18b**. At a slope angle of 5°, when the amount of straw coverage increased from 0.4 kg/m^2^ to 1.2 kg/m^2^, the lateral displacement of the soil decreased by 51.9%. Similarly, at a slope angle of 15°, the reduction was 37.7%. Straw mulching significantly reduces the downslope lateral transport of soil. These findings indicate that the straw effectively counteracts the downslope component of gravity by increasing surface roughness and structural stability.

#### 3.2.3 Interaction effect between straw content, slope and forward farming speed

According to a comparative analysis of **Figures 17**, **18**, it can be concluded that under the same slope condition, an increase in forward velocity leads to a marked reduction in soil displacement. At higher speeds (1.0 km/h), the restraining effect of straw on soil displacement diminishes, indicating that the “cushioning effect” of straw is more prominent during low-speed operations. Furthermore, as the slope increases, the regulatory impact of forward farming speed on soil displacement becomes more pronounced, particularly with respect to lateral displacement.

### 3.3 The impact of straw content on the soil releveling degree

During contour rotary tillage operations, soil tends to move downslope, resulting in a reduction in the initial slope angle and promoting surface leveling. The final slope angle achieved after a single pass of the rotary tillage operation is referred to as soil releveling. In the hilly and mountainous regions of China, repeated rotary tillage practices lead to the downslope translocation of topsoil from upper slopes, resulting in a reduction in the levelness of the surface. This process exposes underlying rocks because of the reduced soil coverage and ultimately decreases the size of the effective cultivable area and compromises agricultural suitability. The results of the above experiments demonstrate that a higher straw content corresponds to reduced soil displacement along the slope and diminished variation in soil surface leveling.

**Figure 19:**
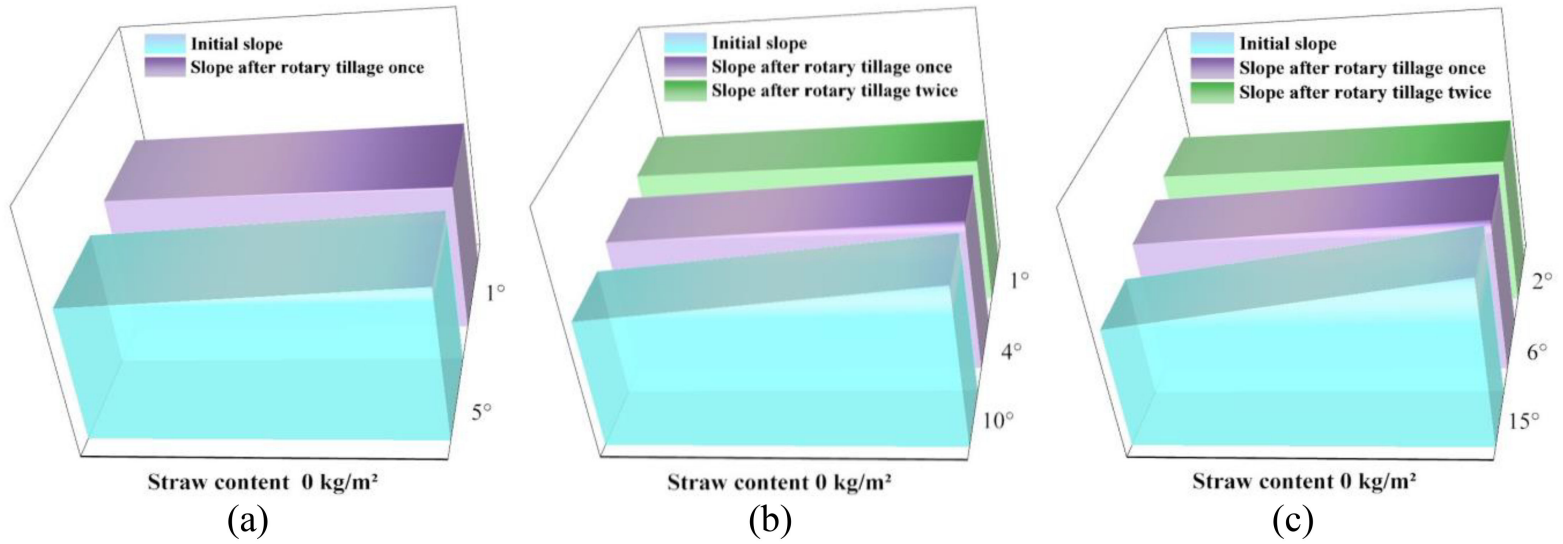
Slope gradient changes during rotary tillage on sloping land without straw cover. **(a)** The slope gradient change after rotary tillage under a slope gradient of 5° without straw cover; **(b)** The slope gradient change after rotary tillage under a slope gradient of 10° without straw cover; **(c)** The slope gradient change after rotary tillage under a slope gradient of 15° without straw cover.

**Figure 20:**
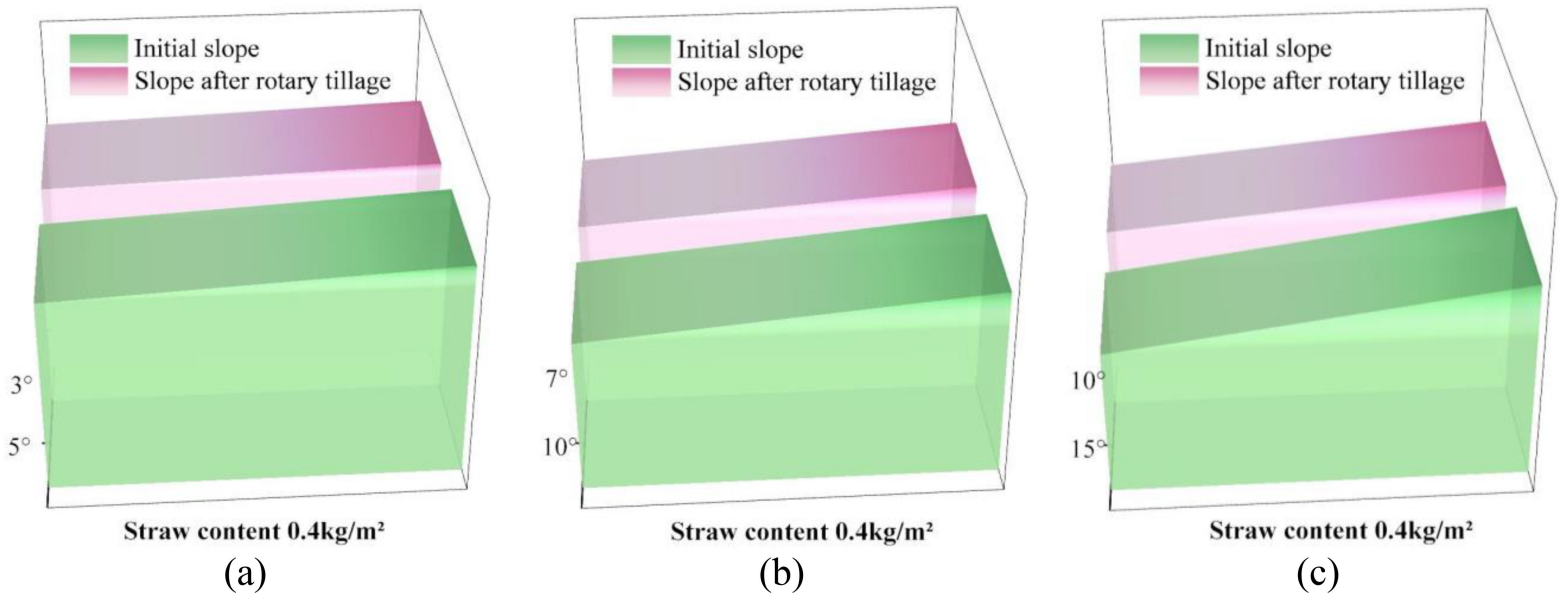
Slope gradient changes during rotary tillage with 0.4 kg/m^2^ of straw cover. **(a)** The slope change of 5° land before and after rotary tillage at 0.4 kg/m²; **(b)** The slope change of 10° land before and after rotary tillage at 0.4 kg/m²; **(c)** The slope change of 15° land before and after rotary tillage at 0.4 kg/m².

On the basis of the aforementioned experiments, an increase in straw content correlates with a reduction in the downslope displacement amplitude of the soil particles, as visually illustrated in **Figures 19**, **20** (0.6km/h,300r/min). A low straw coverage (0.4 kg/m²) resulted in significant downslope soil migration; a moderate to high straw coverage (≥0.8 kg/m²) substantially improved slope morphology preservation and soil surface leveling. Under a slope gradient of 15° and a straw content of 1.2 kg/m², the post-tillage slope angle exhibited minimal variation, indicating that a high straw con-tent effectively maintained the structural stability of the sloped terrain. Previous studies have shown that straw mulching can significantly reduce dust emissions during the plowing process (Chen et al., 2024b), which indirectly reflects the inhibitory effect of straw on soil disturbance. This finding is consistent with the present conclusion that an increase in the straw content leads to a reduction in soil displacement (Chen et al., 2024b, 2022b; Lin et al., 2021).

### 3.4 Orthogonal experiment

#### 3.4.1 Orthogonal experiment design scheme

To further investigate the synergistic effects of the slope gradient, rotary tiller shaft speed, and operational velocity on the horizontal and lateral displacement of soil and to establish a parametric model describing soil displacement in the lateral and horizontal directions during hillslope rotary tillage operations, a three-factor, three-level orthogonal experiment was conducted on the basis of the Box–Behnken de-sign principle with Design-Expert software. The experimental indicators included the horizontal displacement and lateral displacement. The factor coding scheme is presented in **Table 2**. The results of the orthogonal experiment are summarized in **Table 3**, where A, B, and C denote the coded values of the factors.

**Table 2:** Codes of factors.

Encoding	Factors		
	Slope (°)	Forward farming speed km/h	Blade shaft rotational speed r/min
-1	5	0.6	200
0	10	0.8	250
1	15	1.0	300

**Table 3:** Results of orthogonal test.

Serial number	Factors			Horizontal displacement of soil/(cm)	Lateral displacement of soil/(cm)
	Slope/°	Forward farming speed/km·h^-1^	Blade shaft rotational speed/r·min^-1^		
1	10	0.8	250	14.0	6.7
2	10	0.8	250	14.7	6.5
3	10	1.0	200	8.6	4.1
4	5	0.8	200	9.5	4.4
5	15	0.8	200	13.0	6.7
6	10	1.0	300	10.0	6.8
7	10	0.6	200	11.8	7.1
8	10	0.8	250	13.5	6.5
9	5	1.0	250	7.7	4.1
10	15	0.8	300	18.9	8.6
11	10	0.8	250	14.5	6.2
12	5	0.8	300	11.4	5.3
13	10	0.6	300	18.3	8.2
14	5	0.6	250	12.2	6.1
15	10	0.8	250	13.6	6.7
16	15	0.6	250	18.0	9.4
17	15	1.0	250	12.2	6.9

#### 3.4.2 Model establishment and significance test

The outcomes presented in **Table 2** were subjected to variance analysis using De-sign-Expert software, and the analytical results are summarized in **Table 3**. A secondary multiple regression analysis was performed on the experimental data, yielding the following quadratic multiple regression equations for the lateral displacement and horizontal displacement of the soil:

Regression model for soil horizontal displacement (Y_1_), as shown in Equation 8.

(8) $$Y_{1}=14.06+2.66A-2.73B+1.98C-0.325AB+AC-1.27BC-0.2550A^{2}-1.28B^{2}-0.605C^{2}$$

Regression model for soil lateral displacement (Y_2_), as shown in Equation 9.

(9) $$Y_{2}=6.52+1.46A-1.11B+0.825C-0.125AB+0.25AC+0.4BC+0.0975A^{2}+0.2025B^{2}-0.1725C^{2}$$

Analysis of variance was conducted on the two models, and the results are presented in **Table 4**. The results of the significance test indicate that for lateral soil displacement, the P values of the first-order terms A (slope gradient), B (forward farming speed), and C (blade shaft rotational speed) are less than 0.0001, indicating the statistically significant effects of these factors. The F values indicate that the influence of the factors on lateral soil displacement can be ranked as follows: the primary influencing factor is A (F=367.98), followed by B (F= 212.93) and finally C (F=117.1). This quantification conclusively demonstrates that the slope gradient is the predominant natural factor driving the lateral migration of soil in the downslope direction and that the gravitational component parallel to the slope surface serves as the fundamental driving force for soil lateral displacement.

**Table 4:** Variance analysis of orthogonal test result.

Targets of test	Variance source	Quadratic sum	Degree of freedom	Mean square	F	P
Horizontal displacement of soil	Model	167.21	9	18.58	94.62	<0.0001
	A	56.71	1	56.71	288.82	<0.0001
	B	59.40	1	59.4	302.54	<0.0001
	C	30.81	1	30.81	156.91	<0.0001
	AB	0.4225	1	0.4225	2.15	0.1858
	AC	4	1	4	20.37	0.0028
	BC	6.5	1	6.5	33.12	0.0007
	A^2^	0.2738	1	0.2738	1.39	0.2762
	B^2^	6.9	1	6.9	35.13	0.0006
	C^2^	1.54	1	1.54	7.85	0.0265
	Residual error	1.37	7	0.1964		
	Lack of fit	0.2425	3	0.0808	0.2856	0.8343
	Pure error	1.13	4	0.2830		
	Total	168.58	16			
Lateral displacement of soil	Model	33.73	9	3.75	80.6	<0.0001
	A	17.11	1	17.11	367.98	<0.0001
	B	9.9	1	9.9	212.93	<0.0001
	C	5.45	1	5.45	117.1	<0.0001
	AB	0.0625	1	0.0625	1.34	0.2843
	AC	0.25	1	0.25	5.38	0.0535
	BC	0.64	1	0.64	13.76	0.0076
	A^2^	0.04	1	0.04	0.8608	0.3844
	B^2^	0.1727	1	0.1727	3.71	0.0954
	C^2^	0.1253	1	0.1253	2.69	0.1447
	Residual error	0.3255	7	0.0465		
	Lack of fit	0.1575	3	0.0525	1.25	0.4028
	Pure error	0.1680	4	0.0420		
	Total	34.06	16			

In terms of interactions, the P value for BC is 0.0076, which is highly significant; the P value for AC is 0.0535, which is approaching statistical significance. None of the quadratic terms were significant (P > 0.05), indicating that the influence of each factor on soil lateral displacement predominantly follows a linear relationship.

For soil horizontal displacement, the P values of the primary terms A, B, and C are less than 0.0001, indicating their highly significant effects. An analysis of the F values indicates that the influence of the factors on soil horizontal displacement can be ranked as follows: factor B (F = 302.54) is the primary factor, followed by factor A (F = 288.82) and factor C (F = 156.91). This finding indicates that the forward farming speed is the most effective controllable operational parameter for regulating horizontal soil displacement, with a slightly greater influence than the natural slope gradient factor, which is consistent with the conclusions drawn from the single-factor experiments presented in Section 3.1.1. In terms of interactions, the P values for AC and BC are 0.0028 and 0.0007, respectively, both of which are highly significant. Among the quadratic terms, the P values for B² and C² were 0.0006 and 0.0265, respectively, reaching significant or highly significant levels, indicating that the forward farming speed and blade shaft rotational speed have nonlinear influences on the horizontal displacement of soil.

The coefficient of determination (R²) of both models was consistently high, indicating that more than 90% of the variability in the response variable can be explained by the models. These results suggest that the models are suitable for optimization analysis and prediction of operational parameters in sloped land rotary tillage operations.

#### 3.4.3 Analysis of the impact effects of each factor on soil displacement

**Figure 21:**
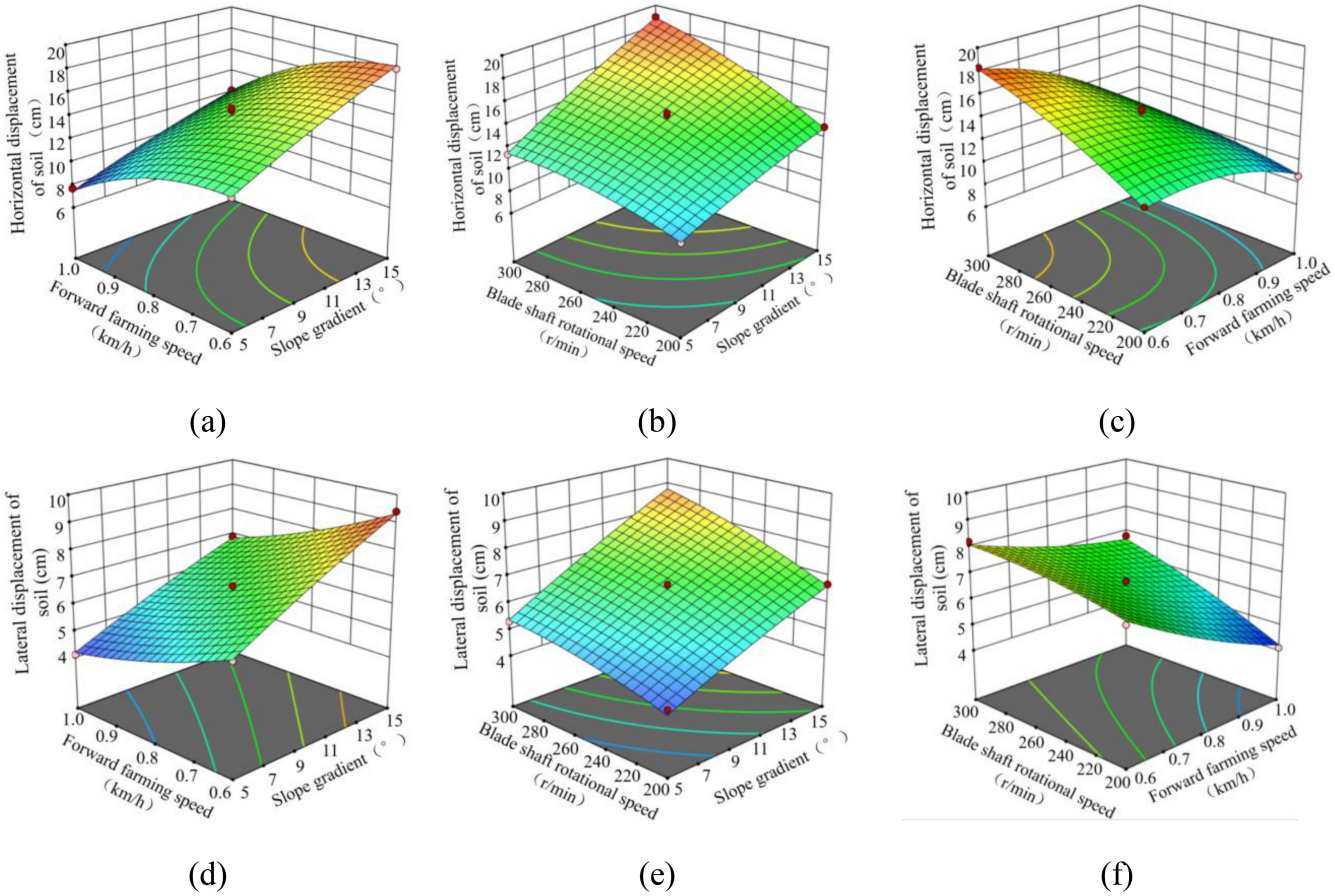
Response surfaces of soil lateral and horizontal displacement. **(a)** The horizontal displacement (forward farming speed and slope gradient); **(b)** The horizontal displacement (blade shaft rotational speed and slope gradient); **(c)** The horizontal displacement (blade shaft rotational speed and forward farming speed); **(d)** The lateral displacement (forward farming speed and slope gradient); **(e)** The lateral displacement (blade shaft rotational speed and slope gradient); **(f)** The lateral displacement (blade shaft rotational speed and forward farming speed).

To visually elucidate the effects of A, B and C and their interactions on soil displacement, a dimensionality reduction method was employed to generate response surface plots (**Figure 21**).

##### 3.4.3.1 Interaction effects analysis

The interaction between the slope gradient and the blade shaft rotational speed (AC) significantly affected the horizontal displacement of the soil (P = 0.0028). The data in **Figure 21b** illustrate that under a high blade shaft rotational speed, an increase in the slope gradient results in a substantial increase in the horizontal dis-placement; conversely, at a low blade shaft rotational speed, the exacerbating effect of the slope gradient on the horizontal displacement remains relatively moderate.

The interaction between the forward farming speed and blade shaft rotational speed (BC) significantly affects both horizontal soil displacement (P = 0.0007) and lateral soil displacement (P = 0.0076). The data in **Figures 21c, f** reveal that under low forward farming speeds, increasing the rotational speed sharply increases the soil displacement. However, as the forward farming speed increases, an increase in the blade shaft rotational speed is associated with a significantly reduced increase in the soil displacement. This finding indicates that increasing the forward farming speed can effectively “dilute” the excessive disturbance to the soil caused by a high blade shaft rotational speed.

##### 3.4.3.2 Factors determining the priority order

On the basis of the F values of each factor in the variance analysis, the primary and secondary sequences of factors influencing soil displacement was determined.

In terms of the factors influencing soil lateral displacement: slope gradient is the primary factor, forward farming speed is the secondary factor, and blade shaft rotational speed is the third factor. In terms of the factors influencing soil horizontal displacement, forward farming speed is the primary factor, followed byslope gradient and blade shaft rotational speed.

The results of the analysis indicate that the forward farming speed is the most critical controllable parameter for controlling the backward (horizontal) dispersion of the soil, whereas the slope gradient is the primary natural factor driving the soil to migrate in the downward-slope direction (laterally). The blade shaft rotational speed impacts both horizontal and lateral soil displacement, but the effect is relatively weak.

##### 3.4.3.3 Suggestions for parameter optimization

To achieve the goal of “low soil erosion and high burial of straw” in sloping land tillage operations, on the basis of the results of the comprehensive orthogonal experiments, the following optimization principles for the operational parameters are proposed. First, the forward farming speed should be gradually increased; within the range permitted by the power of the machinery and the quality of the tillage, increasing the forward farming speed to 0.8–1.0 km/h is the most effective measure to prevent excessive displacement of the soil (especially in the horizontal direction). Additionally, the rotational speed should be determined according to the slope; when operating on steep slopes (with a slope gradient greater than 10°), excessively high rotational speeds should be avoided (the blade shaft rotational speed should be less than 250 r/min) to reduce the lateral soil movement exacerbated by the coupling effect between the rotational speed and slope. Furthermore, balanced operation parameter combinations should be adopted; for example, for moderate slopes (such as 10°), a medium forward farming speed (0.8 km/h) and medium blade shaft rotational speed (250 r/min) should be used. This ensures the quality of the tillage while effectively controlling soil erosion.

## 4 Conclusion

(1) Due to the unclear mechanism of compacted soil movement during cultivation operations on sloping land, which severely hinders the development of specialized rotary tillage machinery for sloping land, in this study, a self-developed sloping land rotary tillage test platform was used to conduct bench tests in hilly and mountainous areas using the tracer method, with the aim of systematically investigating the effects of the slope gradient (5°, 10°, and 15°), blade shaft rotational speed (200–300 r/min), and forward farming speed (0.2–1.0 km/h) on the movement characteristics of compacted soil. The results revealed the regulatory mechanism of the amount of straw coverage (0.4–1.2 kg/m²) on the spatial movement of soil. The results of the bench test revealed that the forward farming speed of the tillage operation is the most effective controllable parameter for suppressing the horizontal and lateral displacement of the soil complex. When the speed is increased from 0.2 km/h to 1.0 km/h, the lateral displacement of the straw is reduced by 65.3%. An increase in the blade shaft rotational speed enhances the migration effect of the complex, especially under steep slope conditions (15°), with the migration toward the lower side of the slope being significantly increased under these conditions. The slope gradient is the dominant natural factor that drives the asymmetric movement of the complex body along the lower side of the slope. When the slope gradient increases from 5° to 15°, the lateral displacement of the straw increases by 166%.

(2) Straw mulching effectively mitigates tillage-induced soil erosion on sloping farmlands by significantly reducing lateral soil displacement. When the straw content increases from 0.4 kg/m² to 1.2 kg/m², the lateral soil displacement is reduced by 51.9% on a 5° gentle slope and by 37.7% on a 15° steep slope. In addition, the use of straw mulching can significantly improve slope releveling. With a high amount of straw coverage, the slope angle changes the least after cultivation, effectively maintaining the stability of the sloped structure. Therefore, appropriately increasing the amount of straw coverage is an important technical approach for controlling soil erosion during slope cultivation operations and increasing the sustainable utilization capacity of the soil.

(3) Through orthogonal experiments and response surface analysis, the order of the primary and secondary influencing factors on the movement of the soil complex were clarified. In terms of the factors influencing soil lateral displacement, slope gradient is the primary factor, forward farming speed is the secondary factor, and blade shaft rotational speed is the third factor. In terms of the factors influencing soil horizontal displacement, forward farming speed is the primary factor, followed by slope gradient and blade shaft rotational speed. Corresponding regression prediction models were also established.

This study systematically revealed the transport mechanism of the straw–root–soil complex under contour tillage conditions on sloping land. This study provides a theoretical basis and methodological support for the design of specialized tillage machinery for sloping land, the optimization of operational parameters, and the quality control of straw in its return to the field.

## Data Availability

The original contributions presented in the study are included in the article/supplementary material. Further inquiries can be directed to the corresponding author.
